# Drug Screening with Genetically Encoded Fluorescent Sensors: Today and Tomorrow

**DOI:** 10.3390/ijms22010148

**Published:** 2020-12-25

**Authors:** Ekaterina S. Potekhina, Dina Y. Bass, Ilya V. Kelmanson, Elena S. Fetisova, Alexander V. Ivanenko, Vsevolod V. Belousov, Dmitry S. Bilan

**Affiliations:** 1 Shemyakin-Ovchinnikov Institute of Bioorganic Chemistry, 117997 Moscow, Russia; bass.dina2014@yandex.ru (D.Y.B.); ikelmanson@gmail.com (I.V.K.); fetisova.el.s@gmail.com (E.S.F.); szd95@yandex.ru (A.V.I.); belousov@fccps.ru (V.V.B.); 2 Center for Precision Genome Editing and Genetic Technologies for Biomedicine, Pirogov Russian National Research Medical University, 117997 Moscow, Russia; 3 Federal Center of Brain Research and Neurotechnologies of the Federal Medical Biological Agency, 117997 Moscow, Russia

**Keywords:** genetically-encoded fluorescent sensors, FRET sensors, in vivo imaging, CRISPR techniques, high-throughput screening (HTS), anti-cancer drug screening, G Protein-Coupled Receptor (GPCR) modulators, Genetically Encoded Voltage Indicators (GEVI), Genetically Encoded Calcium Indicators (GECI)

## Abstract

Genetically-encoded fluorescent sensors have been actively developed over the last few decades and used in live imaging and drug screening. Real-time monitoring of drug action in a specific cellular compartment, organ, or tissue type; the ability to screen at the single-cell resolution; and the elimination of false-positive results caused by low drug bioavailability that is not detected by in vitro testing methods are a few of the obvious benefits of using genetically-encoded fluorescent sensors in drug screening. In combination with high-throughput screening (HTS), some genetically-encoded fluorescent sensors may provide high reproducibility and robustness to assays. We provide a brief overview of successful, perspective, and hopeful attempts at using genetically encoded fluorescent sensors in HTS of modulators of ion channels, Ca^2+^ homeostasis, GPCR activity, and for screening cytotoxic, anticancer, and anti-parasitic compounds. We discuss the advantages of sensors in whole organism drug screening models and the perspectives of the combination of human disease modeling by CRISPR techniques with genetically encoded fluorescent sensors for drug screening.

## 1. Introduction

**Figure 1:**
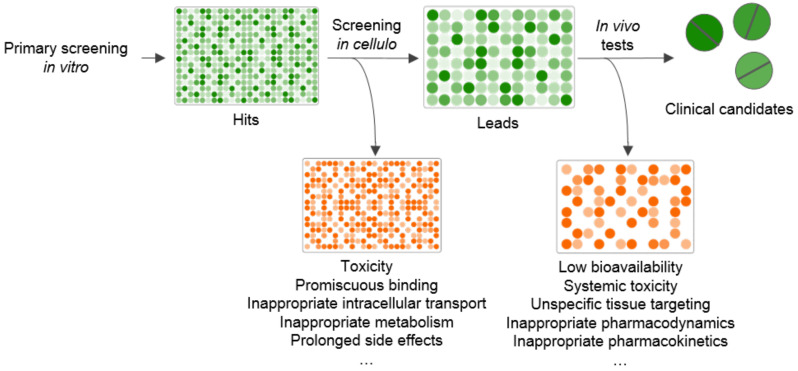
Schematic diagram of drug screening systems. The simpler the initial experimental model is, the more significant proportion of hits is dropped out due to undesired effects in live systems. Green—promising hits, red—dropped out hits.

Assays using genetically encoded fluorescent probes have been extensively developed over the last few decades. This method, combining high scalability and flexibility with specificity, can be applied to various biological systems, from single-cell microscopy to whole-body imaging. The use of genetically encoded fluorescent probes can also accelerate the search for novel drugs, either at the primary screening stage or when confirming hits. Genetically encoded fluorescent sensors allow HTS in cell cultures instead of in in vitro systems, thus providing a more physiologically relevant context for testing drugs. The expression of genetically encoded probes is fully controllable and can be targeted to organelles, cell types, or tissues of interest. Moreover, start, duration, and intensity of expression can also be regulated by the researcher. In most cases, fluorescent biosensors demonstrate low toxicity and do not interfere with normal physiological processes, thus allowing real-time monitoring of live cells instead of end-point assays on fixed cells or cell extracts. They also can be used in more complex in vivo systems to test activity and pharmacodynamics of hits and leads. These features increase the productivity and robustness of drug tests, filtering out artifacts and hits that are active in vitro, but demonstrate low bioavailability, high toxicity, or unspecific effects in live systems (Figure 1). Given that switching from in vitro and in cellulo systems to animal models is the most critical bottleneck in drug discovery, fluorescent HTS on whole organisms seems to be a promising approach in the future. Several methods for modelling human diseases and expressing sensors in animals have been developed, among which CRISPR techniques are thought to be the most specific and efficient.

**Figure 2:**
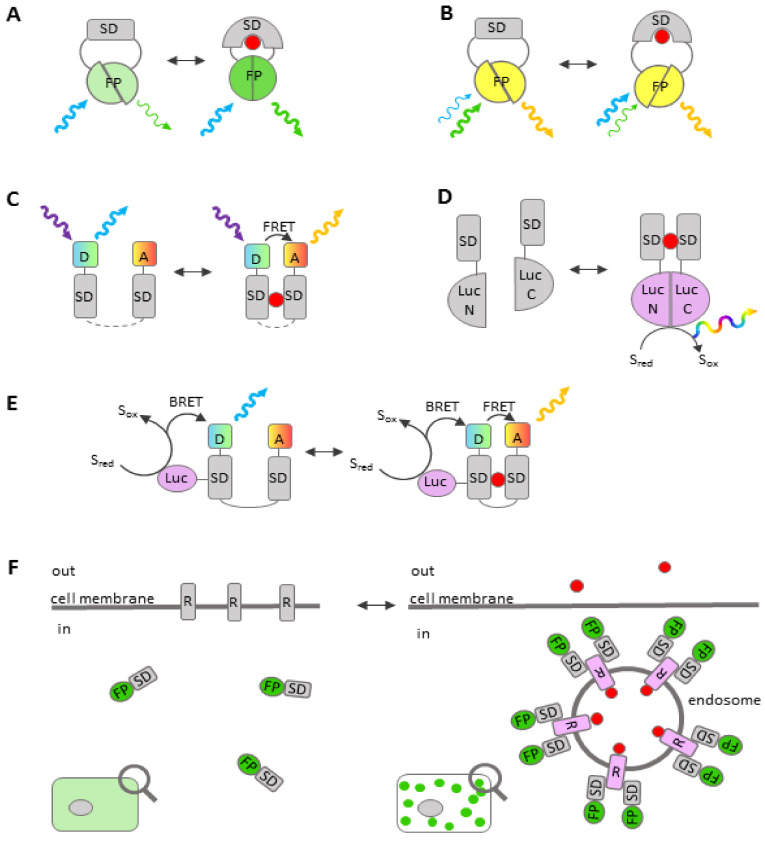
Schematic diagram of fluorescent sensors readouts. (**A**) Single-wavelength ratiometric sensor. After the sensory domain (SD) binds the ligand (red circle), which may be a metabolite, a posttranslational modification, or a protein motif, the conformational change occurs, and the emission of the fluorescent protein (FP) increases. (**B**) Ratiometric sensor based on a single FP. After ligand binding, the conformational change leads to alterations in the absorbance spectrum of the reporter FP. (**C**) FRET-based sensor. In the “open” conformation (or if the subunits of the sensor are separated from each other) resonance energy transfer is ineffective, and only the donor FP (D) fluoresces when excited. In the “closed” conformation, FRET occurs, and the acceptor FP (A) fluoresces when the donor FP is excited. (**D**) Bioluminescent intensiometric sensor. After the ligand binding, the separated N-terminal and C-terminal fragments of luciferase (N-Luc and C-Luc, respectively) arrange an active enzyme capable of converting the reduced substrate (Sred) into the oxidized form (Sox). The wavelength of bioluminescence emitted depends on the substrate. (**E**) HyBRET sensor. (A) luciferase molecule (Luc) is fused to the donor FP and excites it by bioluminescence resonance energy transfer (BRET). Depending on the sensor conformation, the donor FP emits fluorescence or transfers the energy to the acceptor FP. (**F**) Reporter system based on target translocation. When the target molecule is activated (by posttranslational modification, conformational change, etc.), it binds the fluorescent reporter and changes its distribution in the cell. For example, a membrane receptor (R) can recruit the reporter after ligand binding, and, due to the receptor internalization, reporter granules can be observed.

Fluorescent assays yield several types of readout: the intensity of single wavelength fluorescence or bioluminescence, the ratio between different peaks of excitation, the ratio between donor and acceptor fluorescence in a FRET pair (Förster resonance energy transfer), and the lifetime of sensor fluorescence (FLIM). Fluorescence can be excited by an outer light source or by resonance energy transfer from a bioluminescent enzyme (sensors based on BRET—bioluminescence resonance energy transfer and hyBRET—BRET-FRET hybrid biosensors) (Figure 2). Some sensors are suitable for multi-photon excitation, which makes them preferable for whole-body imaging. Depending on the research object, different types of data acquisition are used. In assays based on fluorescent reporter localization, an automated fluorescent microscope is required. If high-resolution imaging is not required, a fluorescence microplate reader is often sufficient. In some cases, e.g., in testing drug resistance in cell populations from patients, flow cytometry may also be used. Thus, fluorescent sensor-based assays are flexible and can be fine-tuned to specific applications. What is more, fluorescent assays are less time- and labor-consuming than most other methods, e.g., radiolabeling or immunochemical staining.

However, there are several points to pay close attention to when using genetically encoded fluorescent sensors, especially in HTS. Heterogeneous and superfluous expression, or improper localization may cause measurement artifacts, sensor toxicity, and other unspecific and undesirable effects. In most cases, cell lines with stable sensor expression are used for HTS. Moreover, monoclonal cell lines may be required for homogeneous expression, especially when using intensiometric sensors. Generally, HTS requires high reproducibility and robustness. These can be characterized by several numeric parameters. The dynamic range of the sensor is the ratio between the parameters (fluorescence intensity, FRET ratio, etc.) of the sensor in the absence of stimulus and the maximal sensor response. The ratio between the mean signal and mean background is often used to characterize assays. However, it does not reflect data variation. The signal-to-noise ratio is defined as the difference between mean signal and mean background normalized to the standard deviation of background. The most commonly used parameter to decide if the assay is suitable for HTS is the Z’-factor, which reflects both dynamic range and data variation. It is calculated based on the means (μ) and standard deviations (σ) of positive and negative controls (c+ and c−, respectively):

$$Z^{\prime }=1-\frac{3\sigma_{c+}+3\sigma_{c-}}{\left|\mu_{c+}-\mu_{c-}\right|}$$

Z′ > 0.5 is required for assays used in HTS to provide reproducible and reliable results [1].

Thus, HTS applications require high reproducibility of sensor signal and expression, restricting the number of systems suitable for tests. However, quite a large number of HTS assays for drug discovery based on genetically encoded fluorescent sensors have been developed. In this review, we briefly describe some genetically encoded fluorescent assays used for the search of modulators of ion channels, Ca^2+^ homeostasis, GPCR activity, and for screening cytotoxic, anticancer, and anti-parasitic compounds. Moreover, we discuss the possibilities for using fluorescent sensors for whole-organism HTS.

## 2. Anti-Cancer Compound Screening

Chemotherapy has developed greatly over the last few decades. However, the existing drugs often lack specificity, causing significant side effects, and the emergence of acquired drug resistance decreases drug efficiency at some point during therapy. Given these considerations, it is especially important to examine the efficacy, specificity, and pharmacodynamics of new drugs in live systems in a high-throughput real-time fashion. Genetically encoded fluorescent sensors meet these requirements, at least for some targets of anti-cancer therapy.

### 2.1. Kinase Inhibitors Screening

Protein kinase-dependent signaling plays a crucial role in the regulation of metabolism, cell cycle, differentiation, and death. The dysregulation of kinase activity is a significant factor in many pathological conditions, including oncological transformation, tumor growth, and metastasis. Therefore, protein kinases are considered a promising target for antitumor drugs. Specific kinase inhibitors are used for mono- or combinational antitumor therapy [2,3,4], but new drugs are required due to the frequent emergence of acquired drug resistance. Moreover, many kinase inhibitors compete with ATP for the binding pocket of the kinase. Since the structure of this pocket is very conservative among protein kinases, competitive inhibitors often lack specificity [5]. Different methods are used to screen the activity of kinases. These include the incorporation of radioactive phosphate containing 32P isotope, the use of phosphorylation-dependent antibodies, the use of non-genetic fluorescent peptide biosensors, and the use of genetically encoded biosensors based on fluorescent proteins [6]. The latter allow real-time monitoring of kinase signaling in cell cultures and tissues in a compartment-specific fashion [6,7,8]. Some genetically encoded fluorescent biosensors can be used not only for single-cell microscopy but also for high-content screening of novel inhibitor libraries.

In different forms of cancer, the tyrosine kinase Src is overexpressed or improperly regulated. This superfluous Src activity is involved in proliferation, adhesion, and invasive behavior of tumor cells [9]. A FRET-based Src indicator was designed and tested in vitro and in HeLa cells. This sensor consists of an Src substrate peptide from the p130cas molecule and a phosphotyrosine-binding SH2 domain from the c-Src molecule sandwiched between ECFP and EYFP. When the sensor is dephosphorylated, it remains in the “closed” conformation and the fluorescent proteins are juxtaposed, allowing energy transfer. After phosphorylation, the new linker conformation separates ECFP and EYFP, increasing the cyan-to-yellow emission ratio. The dynamic range of FRET with this sensor is 43% [10]. It was adapted for evaluating drug efficacy and delivery in the physiological tumor environment using multiphoton excitation and FLIM-FRET microscopy [11]. This approach is less sensitive to the loss of donor emission intensity caused by scattering in tissues [12] and accelerates data acquisition. Under these conditions, the Src indicator was able to mirror the spatial regulation of Src and the pharmacodynamics, delivery, and clearance of the tyrosine kinase inhibitor dasatinib in 3D tumor cultures and intravital tumor xenografts. For instance, in pancreatic ductal adenocarcinoma culture, invasive cell populations demonstrate higher Src activity than the upper surface of the culture. The same pattern was observed in vivo in xenografts, where Scr activity correlated with invasive regions and decreased in the center of the tumor. Dasatinib decreased Scr activity in invasive borders within 50 μm of the vasculature, but the regions in the center of the tumor, and/or more than 100 μm from the vasculature were poorly affected [11].

**Figure 3:**
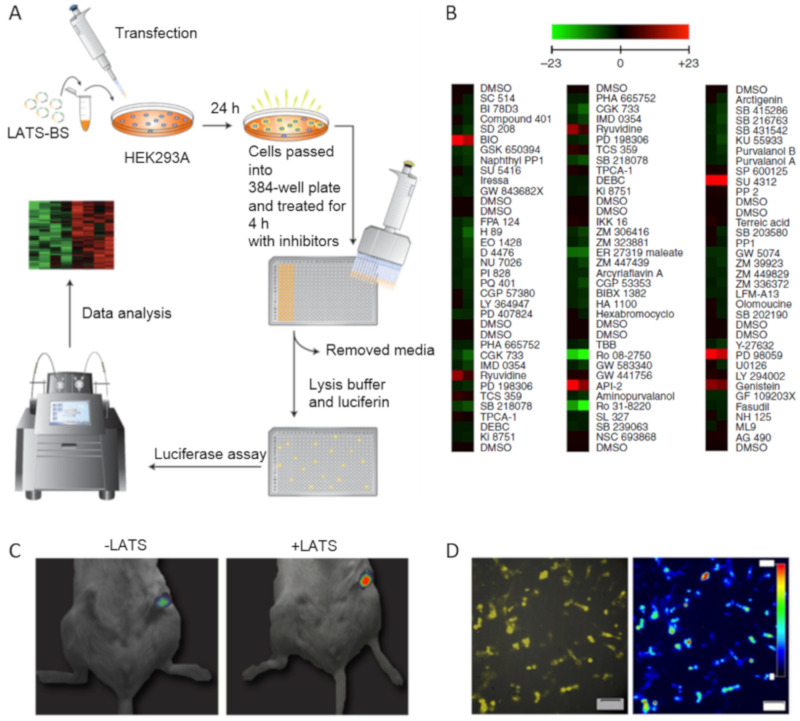
LATS-BS application to several experimental models. (**A**) Experimental design for high-throughput kinase inhibitor screen. Cells were transfected with LATS-BS, treated with a kinase inhibitor library, and luciferase assay was performed on cell lysates. (**B**) Heat map summarizing the results of the kinase inhibitor screen. Drugs that activate LATS-BS are shown in red, drugs that inhibit LATS-BS are shown in green. (**C**) LATS-BS was tested for in vivo imaging. HEK293 cells were transfected with LATS-BS only (-LATS) or LATS-BS and LATS kinase, injected into the mammary fat pad of immunocompromised mice, and bioluminescence was measured. (**D**) LATS-BS was used for bioluminescent imaging of LATS activity in live cells (MDA-MB231 cell line). The figures were taken from Azad et al. with minor changes [14].

The Hippo pathway is involved in organogenesis, differentiation, and regeneration. In this pathway, the Ser/Thr LATS1/2 kinase phosphorylates several effector proteins including the growth-promoting transcriptional co-activator YAP. When phosphorylated on Ser127, YAP binds to cytosolic protein 14-3-3, which prevents YAP from transporting into the nucleus. The inactivation of LATS1/2 or upstream kinases and the amplification of YAP increases cell proliferation and decreases apoptosis and differentiation [13]. The biosensor for LATS (LATS-BS) activity is based on bioluminescence. The minimal YAP fragment capable of interacting with 14-3-3 in a phosphorylation-dependent manner is fused to the N-terminal luciferase fragment, while 14-3-3 is fused to the C-terminal luciferase fragment. When active LATS1/2 phosphorylates the YAP fragment, it binds to the 14-3-3 chimera, and active luciferase is assembled. LATS-BS was successfully used for a small-scale kinase inhibitor screen to identify the upstream regulators of the Hippo pathway. VEGFR, MEK, GSK-3, PKB/Akt, EGF receptor, and CDK 4 inhibitors (SU 4312, PD 98059, BIO, API-2, Genistein, and Ryuvidine, respectively) were shown to activate LATS-BS, while TrkA, SYK, ATR/ATM, CHK1, SGK, and broad-specific inhibitors (Ro 08-2750, ER 27319, CGK 733, PD 407824, GSK650394, and Ro 31-8220 respectively) reduced the LATS-BS signal. Some of these regulators had been previously described. However, the role of VEGFR was established de novo (Figure 3) [14].

The MAPK signaling network affects a wide range of cellular processes, including proliferation, metabolism, and apoptosis. In the MAPK/ERK cascade ERK functions as the final step, reflecting the activity of upstream elements. ERK substrates and final cellular response depend on the type of cell, spatiotemporal regulation, and activity of other signaling pathways. There are several biosensors for ERK activity. The prototype sensor ECAR, consists of mRFP1, a proline-directed WW phospho-binding domain, a peptide from Cdc25C containing the consensus MAPK target sequence, ERK docking peptide, and EGFP [15]. ERK shares the consensus substrate sequence with other MAP kinases [16]. However, the ERK docking motif provides binding and phosphorylation specificity. When phosphorylated, the sensor is in the “closed” conformation, and FRET occurs. Moreover, a CFP-YFP version containing Cerulean and Venus was produced. Both FRET pairs allow 2-photon fluorescence lifetime imaging. The CFP-YFP version allows FRET ratiometric measurement as well, providing almost the same signal-to-noise ratio [15]. After the application of an optimized EEVEE linker backbone, the ratiometric gain of the new sensor (EKAREV) increased fourfold and amounted to 40% [17]. Moreover, EKAREV was transformed into a hybrid FRET-BRET (hyBRET) sensor by fusing a bright Renilla luciferase mutant, RLuc8, to the C terminus of CFP (Turquoise2-GL). In the presence of substrate, the bioluminescence of RLuc8 excites CFP instead of external light sources. The BRET and FRET ratios correlate linearly, and the dynamic ranges in both measurement modes are almost equal. Due to the high signal-to-noise ratio, BRET sensors seem to be reliable instruments for automated drug screening. Indeed, hyBRET-ERK was able to quantify the dose-dependent response to MAPK pathway inhibitors in cancer cell lines cultured in microplates. For example, the IC50 for AZD6244, a MEK inhibitor, was determined using this method [18].

c-Jun N-terminal kinases (JNKs), also known as stress-activated protein kinases are another subfamily of MAP kinases. They are activated by environmental stress signals and cytokines and regulate apoptosis, inflammation, cell differentiation, and proliferation. JNKs are involved in cancer development, neurodegenerative diseases, insulin resistance, diabetes, and heart pathologies, thus JNK inhibitors might serve as drugs in these conditions [19,20,21,22]. JNK activity reporter (JNKAR1) consists of ECFP, forkhead associated domain 1 responsible for phosphoamino acid binding, a substrate sequence linked to the JDP2 docking domain, and Citrine. JNKAR1 is most probably sensitive to JNK1 and JNK 2 isoforms. After specific JNK activation in HeLa cells, the FRET ratio increases by up to 30%. It is noteworthy that the dephosphorylation of the reporter is slower than the dephosphorylation of ATF-2, an endogenous JNK substrate [23]. JNKAR1 has not been validated as an instrument for high-throughput screening. However, given that JNK is a promising drug target, and that the JNK pathway is likely to be a bistable system acting in an all-or-none fashion [23], JNKAR1 might be useful in drug screening assays. Moreover, JNKAR1 has been augmented with an optimized EEVEE linker, and the resulting JNKAR1EV construction demonstrated a dynamic FRET ratio range of about 100% [17].

The activity of the fusion protein Bcr-Abl produced by the Philadelphia chromosome causes chronic myelogenous leukemia (CML). Bcr-Abl is a constitutively active mutant of c-Abl tyrosine kinase. Inhibitors of tyrosine kinases such as imatinib mesylate and dasatinib are the main form of therapy for this condition. However, further mutations in Bcr-Abl make the tumor resistant to these drugs, and thus the development of novel inhibitors is required [24]. Two sensors detecting Bcr-Abl activity in vivo are available. The FRET-based sensor Picchu consists of C-terminally truncated adaptor protein Crk II flanked by CFP and YFP. Crk II Tyr221 is phosphorylated by Abl, EGFR [25], and Bcr-Abl [26] and binds to the SH2 domain, bringing the N-terminal YFP and C-terminal CFP together. The “closed” conformation allows resonance energy transfer from CFP to YFP [25]. The Picchu FRET ratio was demonstrated to reflect the activity of Bcr-Abl and the inhibitory effect and binding kinetics of imatinib and dasatinib. In cells expressing mutant Bcr-Abl forms, the FRET ratio was insensitive to inhibitors. The maximal change in FRET ratio was about 15–20%. However, this was sufficient to distinguish cells with active and inhibited Bcr-Abl by flow cytometry. According to the authors, Picchu can be used for automatic monitoring of drug resistance in mixed population clinical samples as well as for screening of potential next-generation Bcr-Abl inhibitors [26]. It is noteworthy that c-Abl phosphorylates Picchu with the same efficiency as Bcr-Abl [27].

Another sensor for Bcr-Abl activity is called Pickles. Its design exploits the same principles as Picchu. However, the sensitive domain is derived from CrkL, the most characteristic substrate of Bcr-Abl. The FRET pair is m1Venus and circularly permuted ECFP. In the presence of Bcr-Abl, FRET efficiency increases by 80%. Like Picchu, Pickles is phosphorylated not only by Bcr-Abl but also by c-Abl. However, the efficiency of these kinases for Pickles is different. Thus, Pickles is a more specific and sensitive indicator of Bcr-Abl activity. When expressed in cells, it was able to detect the effect, the dose-dependency, and the inhibition rate of Bcr-Abl inhibitors, such as imatinib, nilotinib, and dasatinib. Moreover, this sensor allowed evaluation of the drug response in primary human CML cells [27].

Receptors of growth factors, cytokines, and hormones (RTKs) are a large group of tyrosine kinases. Pathological activity of RTKs is associated with cancer emergence and progression, and the inhibitors of RTKs are widely used as therapeutic agents [28]. The fluorescent reporter of EGFR (epidermal growth factor receptor) activity named EGFRB is based on the specific mechanism of activation of RTKs. When bound to the ligand, RTKs form dimers where the monomers phosphorylate each other, and active receptors are internalized in endosomes. The sensor utilizes receptor clustering and endocytosis. It consists of GFP fused to two SH2 domains from Grb2 adapter protein, which are considered to have high affinity to phosphorylated EGFR. When the EGFRs are inactive, GFP fluorescence is homogeneously distributed throughout the cytosol. After EGFR activation and endocytosis, the sensor binds to the receptors, and fluorescent granules are observed. When expressed in the A549 cell line (human alveolar basal epithelial adenocarcinoma), the sensor demonstrated no response to RTK ligands except for EGF [29]. A549-EGFRB cells were validated as a system for high-content screening of EGFR modulators. After drug administration, the number of fluorescent granules in the wells of a microplate was counted. To measure cell number and cytotoxicity, nuclei stained by DRAQ5 were quantified. The EGFRB system demonstrated a high signal-to-noise ratio (21:1) and reproducibility. Using this system, a library of 6912 Food and Drug Administration-approved and known bioactive compounds was screened, and 12 of 13 reported EGFR kinase inhibitors from this library were picked as positive. Moreover, the Hsp90 inhibitor 17-DMAG was shown to decrease EGFR activity, most probably because Hsp90 is necessary for EGFR maturation. In the same screen, confirmed EGFR activators were shown to increase the number of granules. What is more, A549-EGFRB cells were successfully used for a dose-response test of EGFR modulators picked from the previous screen. According to the authors, EGFRB assay could be a potent instrument in EGFR modulator discovery [30].

### 2.2. Transcription Factors Regulators Screening

P53 takes part in the regulation of crucial cellular processes, including metabolism, cell cycle, stress response, and apoptosis. Decreased p53 activity due to mutations, misregulation, or enhanced decay is associated with tumorigenesis and cancer progression. Furthermore, p53 is ubiquitinylated and targeted to degradation by hDM2 protein, which is overexpressed in some types of cancer [31]. In order to find novel p53-hDM2 interaction inhibitors, a system for automated high-content screening for protein-protein interaction disruptor (PPID) was established. First, p53 was fused to GFP and augmented by an NLS localizing the construction in the nucleolus. hDM2 carried both NLS and NES and was fused to RFP. Naturally, both proteins colocalized in the nucleolus. In the presence of the known p53-hDM2 interaction inhibitor Nutlin-3, hDM2 was exported to the cytoplasm. A library of 220,000 small-molecule compounds was screened, and the assay demonstrated high reproducibility. However, a thorough analysis of fluorescence artifacts and cytotoxic effects was required to omit false-positive hits. Finally, three compounds related to methylbenzonaphthyridin-5-amine were confirmed to increase p53 protein level and apoptosis and to cause cell cycle arrest and growth inhibition in a p53-dependent fashion [32].

The gene c-Myc plays key roles in cell cycle regulation, growth, differentiation, and apoptosis. In the vast majority of cancers, this protein is abnormally abundant, stable, and active, which makes it a potential anti-cancer drug target. Mitogens and other stimulators activate c-Myc via Ser62 phosphorylation by ERK kinase. After stimulator removal, Ser62 recruits glycogen synthase kinase-3β (GSK-3β), which phosphorylates the Thr58 residue and targets c-Myc for proteasomal degradation [33]. c-Myc activation sensor consists of the c-Myc activation motif fused to the N-terminal domain of Renilla luciferase and GSK-3β phosphoamino acid binding domain fused to the C-terminal domain of *Renilla luciferase*. When c-Myc activation motif is phosphorylated, it binds to GSK-3β fragment, and the split luciferase fragments form an active enzyme. The sensor was tested in murine xenografts where it was able to reflect drug impact on c-Myc phosphorylation [34]. The sensor also proved to be applicable to high-throughput screening of c-Myc inhibitors. A library of 5000 compounds was tested on SKBR3 cells (breast cancer cell line with c-Myc overexpression), and about 1% of compounds were identified as clean positive hits, including known c-Myc pathway inhibitors, and nitazoxanide, a widely used antiprotozoan drug with few side effects. It should be mentioned that cell proliferation and luciferase activity inhibitors were also identified as positive hits. After further validation in c-Myc-associated cancer cell lines and xenografts, the c-Myc inhibiting and antineoplastic effects of nitazoxanide were confirmed [35].

### 2.3. Cell Death Signaling Inducers Screening

Another approach in anti-cancer drug discovery is the search for inducers of certain types of cell death, like apoptosis, mitotic catastrophe, or immunogenic cell death, irrespective of drug target. Some of these conditions can be detected using immunochemistry or chemical labeling. However, these methods are time- and labor-intensive and usually require fixation and permeabilization of cells. Thus, they are not well suited for high-throughput and in vivo assays. However, these phenomena can be observed in automated systems using genetically encoded fluorescent reporters.

For example, a system of two fluorescent reporters of mitotic catastrophe has been developed. Histone H2B-GFP chimera stably expressed in HCT 116 cells (human colon carcinoma) provides chromosome tracking, while *Discosoma striata* red fluorescent protein fused to centrin (DsRed-Centrin) visualizes the centrosomes, allowing polyploidy and multipolar divisions to be monitored. After plotting mean nuclear density against nuclear heterogeneity of H2B-GFP fluorescence, cells arrested in metaphase were observed as a distinct population, while other cells, including apoptotic ones, were distributed all over the plot. The assay was able to reflect mitotic catastrophe induction by several mitotic blockers, distinguish different cell fates after treatment, and show different susceptibility to the treatment in wild-type and p53^−/−^ HCT cells. It is suitable for the microplate format and can be used for high-throughput detection of mitotic catastrophe [36].

The activation of effector caspase-3 is one of the most critical steps of apoptosis [37]. A FRET-based reporter of caspase-3 activity has been created. It consists of CFP, YFP, and a linker sequence containing the caspase-3 recognition site. After caspase-3 activation caused by UV radiation, toxic compounds, and other apoptotic stimuli, the sensor protein is cleaved, and the FRET ratio decreases. FRET ratio does not decrease in necrotic cell death. When expressed in HeLa-C3 cells grown in microplates, the sensor was able to dose-dependently reflect the pro-apoptotic effect for several compounds of known biological activity, such as vincristine, paclitaxel, and hydroxyurea, as well as for some novel plant-derived substances [38]. It should be mentioned, however, that YFP spectral properties are highly dependent on H^+^ and Cl^−^ ion concentrations, and the latter may dramatically change during apoptosis. Venus fluorescent protein is more stable and can be used in such FRET-based sensors instead of YFP [39]. Moreover, the cleavage site DEVD, used in [38], can be recognized by other caspase-3-like proteases (DEVDases), for example, caspase-7 [40].

Another approach for detection of caspase-3-like protease activity is using switch-on fluorescence-based caspase-3-like protease activity indicator (SFCAI). This sensor is based on a circular permutant of Venus protein, whose native N- and C-termini are connected by DEVDase recognition sequence. The artificial N- and C-termini are fused to Npu DnaE intein from Nostoc punctiforme, which catalyzes the trans-ligation of the termini, forming a cyclic protein. Cleavage by DEVDases alters the protein conformation and enables fluorescent activity. The sensor was tested in several cell cultures, and it was able to specifically reflect apoptotic DEVDases activation by cisplatin in a real-time and dose-dependent fashion. It is noteworthy that MCF-7 cells (human breast cancer) turned out to be the most convenient for fluorescent microscopy because they did not detach from the culture platform. The sensor also detected apoptosis in 3D cell cultures in soft agar. A group of relative sensors was developed based on different fluorescent proteins: superfolder GFP, Cerulean, and mCherry. According to the authors, SFCAIs could be used in high-throughput screening assays for pro-apoptotic agents due to their high sensitivity and robust signal [40].

Some apoptotic cells undergo membrane rupture after caspase activation entering secondary necrosis [41], so caspase activity reporters can leak from the cytoplasm, causing non-specific shifts in sensor readout. To distinguish between apoptosis and primary and secondary necrosis, one can use an additional fluorescent protein localized in membrane organoids, which stay intact during necrosis. For example, mitochondrion-targeted red fluorescent protein can be used together with a cleavable FRET-based caspase-3/7 activity reporter containing ECFP and EYFP. In apoptotic cells, the FRET ratio decreases, and in necrotic cells the FRET signal is lost, while RFP fluorescence remains constant. For cells stably expressing both reporters, high-throughput adaptable protocols for live-cell imaging and flow cytometry analysis are available [42].

Immunogenic cell death (ICD) inducers are another type of anti-cancer drugs. They stimulate cancer cells to emit signals that attract and activate immune cells. During premortem stress several processes occur, for example exposure of calreticulin on the surface of the cell, release of ATP, and the exodus of high-mobility group box 1 (HMGB1) protein from the nucleus to the cytoplasm [43]. LC3 protein migrates to autophagy-specific granules [44]. Using U2OS cells (human osteosarcoma) expressing calreticulin fused to RFP and HMGB1 or LC3 fused to GFP, a library of more than 500 compounds was screened. Cell nuclei were stained with DAPI, and nuclear pyknosis was also detected. This screening demonstrated that some tyrosine kinase inhibitors can induce ICD, which was further confirmed in cell cultures and murine xenografts [45]. HMGB1 exodus can also be observed via the “retention using selective hooks” (RUSH) system. In this system, U2OS cells express streptavidin fused to the NLS3 sequence and HMGB1 fused to streptavidin binding protein (SBP) and GFP. In the absence of biotin, the HMGB1-SBP-GFP chimera is bound to the streptavidin-NLS3 construction and localized in the nucleus. However, after ICD inducers and biotin treatment, GFP fluorescence is also observed in the cytosol. The assay is performed on fixed cells. It has been validated for high-throughput screening for ICD inducers. The RUSH system allows comparison of cell images with and without biotin treatment, thus filtering out false-positive results detected due to drug fluorescence and other unspecific factors [46]. This approach can also be applied to the search for protein secretion inhibitors [47].

The LC3-GFP reporter can be used in search of caloric restriction mimetics (CRM) as an autophagy reporter in tandem with protein acetylation monitoring. CRM can act as cardio- and hepatoprotectors and stimulate the immune response against tumor-associated antigens. LC3-GFP was used to identify CRM in a library of 200 compounds, and positive hits were tested for cytotoxicity and protein acetylation state. The screening revealed a CRM effect of 3,4-dimethoxychalcone, which was further confirmed to improve the T-cell dependent effect of the chemotherapeutic drug mitoxantrone [48].

### 2.4. Energy Metabolism Modulators Screening

Another promising area to target for anti-cancer anti-proliferative drugs is ATP production. Most cancer cells are characterized by metabolic changes, the Warburg effect is manifested as the predominance of glycolysis over oxidative phosphorylation [49]. The identification of small molecular inhibitors of glycolytic ATP production may be leveraged in the creation of some anti-cancer drugs. Imamura et al. developed ATeams FRET sensors for intracellular ATP level detection based on the epsilon subunit of *Bacillus subtilis* FOF1-ATP synthase fused to cyan fluorescent protein (mseCFP) at the N-terminus and yellow fluorescent protein (cp173-mVenus) at the C-terminus [50]. The physiological role of the ε-subunit is thought to be regulation of FOF1-ATP synthase activity depending on the intracellular ATP level. The subunit binds to ATP but does not hydrolyze it. In the ATP-bound form, the subunit retracts to draw the two fluorescent proteins close to each other, increasing FRET efficiency. ATeam version AT1.03 demonstrated a FRET signal dynamic range of about 2.5, and the detectable ATP concentration range was 1–8 mM. In 2018, Zhao et al [47]. designed a three-step assay protocol in 96-well fluorescent plate reader format based on long-term transfectant K562 cells expressing ATeam FRET sensor version AT1.03, which allowed identification of compounds inhibiting glycolytic or OXPHOS-dependent ATP production, or both [51]. It was demonstrated that using 105 cells/well in 96-well fluorescent plate reader format made the variability of sensor FRET ratio between wells insignificant and the detected signal reproducible. The first read allowed detection of drugs that inhibit glucose-independent oxidative phosphorylation-dependent ATP production because measurements were made in the absence of glucose. The authors demonstrated that in those conditions K562 cells had produced ATP by OXPHOS using stored substrates for up to 3 h or until an inhibitor had been added. The second read was performed after 20 min incubation with 5 mM azide to block respiratory complex IV and provided an opportunity to select compounds that affect FRET ratios at the 1st read via fluorescence artifacts. Subsequent incubation with 20 mM D-glucose for 20 min preceded the third read. In this read, glycolysis inhibitors preventing ATP synthesis were detected. Unfortunately, the described method was not able to identify compounds that inhibit glycolytic or OXPHOS-dependent ATP production with a delayed mechanism of action. However, this FRET screening made it possible to test 813 compounds in the NCI’s (National Cancer Institute) Mechanistic Set III. ATP level influence was confirmed by luciferase ATP assay. The assay identified 14 compounds inhibiting oxidative phosphorylation-dependent ATP production by >75% and 13 compounds inhibiting glycolysis by >80%. Three of the detected inhibitors of oxidative phosphorylation, nonactin (NSC 52141), usnic acid (NSC 5890), and peliomycin (NSC 76455), were described previously. Other inhibitors were identified for the first time. ATeam FRET screening may be a basis for selecting inhibitors of ATP production with the ability to differentiate the path of ATP synthesis inhibition, i.e., glycolysis or OXPHOS. Significantly, Zhao et al [47]. showed that the identified inhibitors of oxidative phosphorylation were not more antiproliferative than the NCI’s compound library as a whole, whereas glycolysis inhibitors were significantly more effective.

Due to the Warburg effect, many cancer cells show significantly lower NAD+/NADH ratios than non-cancer cells. The genetically encoded SoNar sensor was constructed by insertion of cpYFP between two subunits of NAD(H) binding protein Rex from *Thermus aquaticus* (T-Rex). NAD+ saturation of SoNar increases fluorescence exited at 485 nm, while NADH binding increases fluorescence exited at 420 nm. SoNar can specifically bind NAD+ or NADH (Kd ≈ 5.0 μM and ≈ 0.2 μM, respectively, at pH 7.4). It is a pH-stable, effective ratiometric sensor for NAD+, NADH, and their ratio. SoNar displays an 8-fold dynamic range in living cells. Zhao et al [47]. presented SoNar as an excellent choice for HTS of the NAD+/NADH ratio in living cancer cells with drug screening opportunities [52]. More than 5500 unique compounds from the commercially available small-molecule libraries that might affect cellular metabolism were screened in the microplate format in H1299 cells using the SoNar sensor. Seventy-eight compounds were identified that significantly decreased the NAD+/NADH ratio in H1299 cells, and most of them did not exhibit obvious H1299 cell toxicity. Just 9 of 78 compounds were toxic. Compounds that significantly increased the NAD+/NADH ratio in H1299 cells exhibited H1299 cell toxicity (8 of 12 identified), among which were β-lapachone, shikonin, and fascaplysin, potent widely studied anti-tumor agents. The Akt inhibitor, also known as KP372-1, was identified as the compound with the most pronounced influence, increasing NAD+/NADH ratio, and with high cytotoxicity at 100 nM concentration for cancer cells of different origin, and with low toxicity toward various primary cells. KP372-1 was tested in H1299 xenografts expressing SoNar in mice, and the suppression of tumor growth was confirmed. Moreover, KP372-1 was shown to be cytotoxic to cancer cells independently from Akt inhibition. KP372-1 was proven as a potent NQO1-mediated redox cycling agent that causes severe oxidative stress and induces cancer cell apoptosis. The pronounced antitumor effect, bioavailability, and pharmacokinetics make KP372-1 a promising candidate for antitumor drugs.

## 3. G Protein-Coupled Receptor (GPCR) Modulator Screening

Signaling pathways triggered by GPCRs are a major group of drug targets [53]. To enable high-throughput and high content screening of GPCR pathway modulators, sensors reflecting enzyme activities and second messenger concentrations have been developed.

The AKAR family of protein kinase A (PKA) activity sensors is based on FRET. AKAR3 consists of ECFP, forkhead associated domain 1 (FHA1) capable of phosphoamino acid binding, optimized PKA substrate peptide, and YFP (Citrine). It was examined as an instrument for microplate screening. The sensor demonstrated a dynamic range of about 30% and high sensitivity and reproducibility. Since cAMP affects not only PKA activity, a reporter of cAMP concentration has also been established. This reporter, named ICUE1, consists of full-size Epac1 protein sandwiched between ECFP and citrine and exploits the conformational change in Epac1 after cAMP binding. The optimized ICUE3 version was tested as a microplate assay complementary to AKAR3 measurement. HEK293 cells expressing AKAR3 or ICUE3 were treated with a library of 160 compounds of known physiological activity to identify activators of the PKA signaling pathway. After this treatment, the standard agonist (isoproterenol) was added. Cells pre-treated with PKA pathway inhibitors demonstrated a weaker response than others. From this library, all three known β-adrenergic agonists (isoproterenol, ritodrine, and epinephrine) increased the FRET ratio of AKAR3. The β-adrenergic antagonist propranolol decreased the response to isoproterenol both in AKAR3 and ICUE3 expressing cells. However, bilirubin was shown to decrease the ICUE3 response to isoproterenol, but not the AKAR3 response, suggesting that bilirubin affected cAMP production, but not PKA activity. According to the authors, AKAR3 is more sensitive to agonists than ICUE3, because one active PKA molecule is able to phosphorylate many sensor molecules [54]. It should be mentioned that AKAR3 was further augmented with the optimized EEVEE backbone, which increased the dynamic range of the sensor up to 60% [17].

The cAMP biosensors are quite numerous. Most of them are based on FRET and contain the whole Epac protein or fragments of it as sensor domains because they respond to cAMP concentration changes much faster than sensors based on cAMP binding domains from PKA, for example, Epac2-camps consists of CFP, cAMP-binding domain B of Epac2, and YFP [55]. Epac2-camps expressed in B16F10 (murine melanoma) cells via the BacMam system were shown to reflect the activation of the Gs-coupled melanocortin-1 receptor MC1R by different agonists. The Z′ factor for specific agonists was >0.6, demonstrating that the BacMam-Epac20camps system might be used in high-content screening of receptor modulators [56]. The use of FPs with advanced spectral properties can improve the reliability and robustness of the assays. As an example, the sensor named TEPACVV consists of mTurquoise, a catalytically dead fragment of Epac, and a tandem of Venus proteins. Due to the enhanced brightness and single-exponential fluorescence decay of mTurquoise, TEPACVV is suitable both for FLIM and ratiometric measurements. When expressed in HEK293 cells, the sensor demonstrated a ~30% increase in mTurquoise fluorescence lifetime and a ~80% decrease in FRET ratio after the addition of adenylyl cyclase activator forskolin and phosphodiesterase inhibitor IBMX [57]. A protocol for microplate cAMP assay utilizing BacMam-delivered Epac20camps or TEPACVV sensors is available [58].

The results of GPCR activation depend on the spatial and temporal patterns of signaling. A growing body of evidence suggests that different ligands trigger intracellular cascades which differ in duration, localization, and balance between signaling branches. Thus, multiplex sensor systems capable of reflecting multiple pathways could increase the efficiency of drug screening. For instance, a system for simultaneous detection of phosphatidylinositol 4,5-bisphosphate (PIP2) and diacylglycerol (DAG), the substrate and product of phospholipase C (PLC) activated in Gq pathway, has been developed. DAG biosensors consist of cpGFP fuzed to protein kinase Cδ. There are two biosensors, Upward DAG2 and Downward DAG. The former demonstrates increased fluorescence when bound to DAG, and the latter is brighter in the free state. The in cellulo dynamic range of Upward DAG2 biosensor is higher than in Downward DAG biosensor (about 100% and 40%, respectively). PIP2r sensor consists of two monomers of dimerization-dependent red fluorescent protein fused to pleckstrin homology (PH) domains from PLCδ. PIP2 recruits the PH domains to the membrane, enabling dimerization-dependent proteins to dimerize and become fluorescent. PIP2r and DAG biosensors can be coexpressed in cells. The system is suitable for automated microplate assays and enables fine detection of signaling patterns [59].

G-proteins can become desensitized after a long period of agonist exposure, thus in some cases it may be preferable to directly detect the interaction between the GPCR and its ligand. A system based on GPCRs carrying a nuclear localization sequence (NLS) incorporated into a conformation-dependent site in helix 8 is suitable for this purpose. NLS-GPCRs are trafficked to the cell membrane and then translocated to the nucleus; however, binding of agonists and antagonists leads to retention of the receptor on the cell surface. The incorporation of NLS has been shown not to change the structure of the ligand-binding site [60]. The density of the receptors on the cell membrane is quantified via enzyme fragment complementation technology [61]. A fragment of β-galactosidase is fused to the N-terminus of the target NLS-GPCR. When NLS-GPCR is localized on the membrane, this fragment is extracellularly oriented and can be cleaved by thrombin. The cleaved fragment associates with the complementary part of β-galactosidase, and the catalytically active enzyme hydrolyzes a luminogenic substrate. This system has been shown to reflect the binding of agonists and antagonists in a concentration-dependent fashion, and to be compatible with several types of GPCR (D1 and D5 dopamine receptors, HT1B and 5HT1A serotonin receptors, μ-opioid receptors, M1 muscarinic receptors, and β 2-adrenergic receptors) expressed in HEK 293 cells. According to the authors, it is suitable for microplate high-throughput screening of GPCR modulators and demonstrates high reproducibility and signal-to-noise ratio. Moreover, this approach may be used for the search of orphan GPCR ligands [62].

However, it should be mentioned that the effect of GPCR modulators may significantly differ in different model systems. The set of receptor partners, signaling molecules, and effectors depends on cell type, individual cell heterogeneity, microenvironment, and other factors. Commonly used cell cultures, like HEK 293 cells, are often physiologically irrelevant. Given these considerations, the use of primary cultures or IPCs-derived cultures might decrease the attrition rate of drug screening assays [63].

## 4. Membrane Potential Modulator Screening

### Genetically Encoded Voltage Indicators (GEVI)

The human genome contains more than 300 genes encoding ion channels [64,65], almost half of which are voltage-gated, i.e., activated by changes in membrane potential [66]. Voltage-gated ion channels (VGCs) play crucial roles in many cellular processes and are particularly important for the functioning of electrically excitable cells such as neurons and cardiomyocytes. This places them among the key drug targets for neurological and cardiological disorders and is why the development of high throughput screening methods for VGC inhibitors and modulators is important.

The drug-VGC interaction strongly depends on the VGC conformational state, which in turn depends on transmembrane potential [67]. Drug-screening assays targeting VGCs must therefore include means to control and measure membrane voltage. The gold standard technique enabling this task is voltage-clamp, however this method is labor-intensive and slow, and cannot be applied in high throughput approaches. The available higher throughput methods have other intrinsic limitations. Multielectrode arrays do not provide single-cell resolution and are insensitive to sub-threshold membrane potential changes. Automated planar-array patch-clamp platforms are much more productive than manual patch-clamp but produce lower quality data and still have reduced throughput compared to fluorescent assays. They are also expensive and are not very compatible with neurons [68].

The development of voltage sensitive dyes and, later, genetically encoded voltage indicators (GEVIs) and optogenetic actuators (reviewed in [69,70,71,72,73]) opened opportunities for all-optical electrophysiology. An important step towards this goal was made by Hochbaum et al. when they developed the improved voltage indicators QuasAr1 and QuasAr2 on the basis of Archaerhodopsin 3 (Arch) from *Halorubrum sodomense* [74]. Archaerhodopsins contain retinal chromophore that exhibit near-infrared fluorescence in response to membrane depolarization due to Schiff base protonation. This mechanism allows for sub-millisecond response kinetics, allowing resolution of individual spikes in mammalian neurons, and a large voltage sensitivity [71]. However, wild-type archaerhodopsins cannot serve as voltage sensors since they produce a proton-pumping photocurrent in response to excitation light. They are also very dim, and their brightness depends non-linearly on illumination intensity. Five rounds of random mutagenesis and rational design allowed Hochbaum and colleagues to create non-pumping QuasArs (Quality superior to Arch) with improved kinetics and sensitivity, linear optical response, and enhanced, although still extremely far from perfect, brightness. Together with red-shifted spectra and high photostability these beneficial properties put QuasArs, developed in 2014, among the best GEVIs for in vitro studies [75]. In a complementary addition to QuasArs, Hochbaum et al. engineered a novel blue-shifted channelrhodopsin CheRiff and combined it with QuasArs in the bicistronic vectors Optopatch1 and Optopatch2. The important advantage of these actuator/indicator pairs was their spectral orthogonality—the high sensitivity and optimized spectra of both CheRiff and QuasArs allow their combined use with negligible optical crosstalk [74].

The first application of the Optopatch platform in pharmacological research was screening for blockers of Nav1.7, a voltage-gated sodium channel implicated in pain [76]. For this purpose, HEK293 cells stably expressing CheRiff and QuasAr2 were created. The cells also expressed Nav1.7 and inward rectifier potassium channel Kir2.1, which gave them the ability to produce regenerative electrical spikes [77,78,79]. The resulting Nav1.7 Optopatch Spiking (Nav1.7-OS) HEK cells were used for probing Nav1.7 pharmacology. Simultaneous optical and patch-clamp recording confirmed that CheRiff stimulation allows accurate manipulation of the membrane potential, inducing well-reproducible action potentials or subthreshold depolarization depending on excitation light intensity. The authors reproduced traditional voltage clamp protocols, varying the duration and intensity of the depolarizing blue light pulses, and conducted a test high-throughput screening of activity-dependent Nav1.7 modulators (potential analgesics) from 320 FDA-approved compounds. The screening was carried out in the 384 well plate format, approximately 150 cells were recorded in each well. Automated scanning of the entire plate required 20 min. The assay produced a 12.2% hit rate where hits were defined as compounds with δ greater than 5 standard deviations from the average of negative controls, the Z′ factor of the assay had an acceptable value of 0.57.

Since sodium channel inhibitors are known for their promiscuity [80], the hits were re-tested using the same Optopatch Spiking platform for their ability to interact with the cardiac Nav1.5 channel. None of the newly discovered inhibitors showed subtype selectivity, in contrast to the specific Nav1.7 blockers PF-04856264 and TTX that were used as controls. The off-target binding made the hits unusable for use as analgesics due to cardiac unsafety. Thus, the assay did not produce new specific Nav1.7 drugs, however it demonstrated the potential of all-optical electrophysiology in drug screening.

The CheRiff actuator utilized in the Optopatch platform enables sufficient transmembrane voltage control for many screening applications. However, unlike the traditional voltage-clamp technique this optogenetic control is unidirectional, i.e., it only enables depolarization of the cellular membrane. Streit and Kleinlogel [67] proposed a light-induced electrophysiology (LiEp) approach allowing both depolarizing and hyperpolarizing optically-controlled voltage steps. For this purpose, they combined two actuators: a blue light-gated cation channel ChR2 (the depolarizer) [81] and a yellow light-driven outward proton pump ArchT (the hyperpolarizer) [82]. The resulting fusion construct enabled bi-directional optical control of the membrane potential with millisecond resolution. In proof-of-concept assays the LiEp platform was successfully tested in combination with different optical voltage indicators, including genetically encoded QuasAr1 [74]. The all-optogenetic system allowed accurate tuning of Nav1.5 channel conformational states and allowed drug-channel interactions to be reported with high precision, comparable to that of parallel voltage-clamp recordings. However, the use of the assay was limited by almost complete overlap between the QuasAr1 and ArchT excitation spectra. This overlap restricted the tunability of the system, making it necessary to employ yellow laser illumination both for hyperpolarization and voltage imaging, and superimpose ChR2-activating blue light pulses to depolarize the membrane to the required potential.

Low brightness is the common weak point of all opsin-based GEVIs including QuasArs [83]. This drawback seriously hinders their applicability in cell-based assays and especially in in vivo studies due to the high illumination intensities required for sensor illumination. One aspect of this problem is that the high (up to 400 W/cm^2^ and higher) red light power needed for QuasAr2 excitation induces notable heating in the small volume of a 384-well plate [84]. Low brightness can be compensated by fusing the opsins to a bright fluorescent protein (FP) and using the resulting construct as an electrochromic FRET sensor, in which FP serves as a donor and opsin as an acceptor of fluorescence [85]. The wide absorption spectra of microbial rhodopsins is compatible with bright FPs of different colors, whose donor fluorescence is used as readout. Unfortunately, such eFRET sensors are far less sensitive than their parental opsins, and they lose the advantage of red-shifted excitation that made the parental opsins compatible with optogenetic actuators and better performing in situations where high signal-to-noise ratios are crucial [75].

However, a large proportion of drug-VGC interaction assays do not actually require voltage control at the single-cell resolution provided by voltage-clamp or optogenetic actuators. One of the rapidly developing drug-screening approaches is the use of human induced pluripotent stem cell-derived cardiomyocytes (hiPSC derived CMs). HiPSC derived CMs offer huge opportunities in cardiac research, enabling modeling of heart diseases and testing the therapies directly on human (and even patient-specific) cardiomyocytes in well-reproducible and highly scalable experiments. Cultured cardiomyocytes form a spontaneously contracting syncytium of cells, connected via gap junctions. This electrically interconnected monolayer can be paced externally by precise optogenetic or rough field stimulation of only a subset of cells in the well or dish. Regardless of the technique used for pacing, all cells in the syncytium will generate action potentials (APs) and demonstrate other CM-specific alterations of membrane potential, whose parameters such as AP duration or the occurrence of early afterdepolarizations (EADs), can be recorded with a high throughput and at the single cell resolution using GEVIs.

High-throughput optical recoding of the cardiomyocyte AP waveform is in particular demand for cardiac safety evaluations. Non-specific off-target effects on VGCs often preclude the success of the new drugs, mainly due to a pro-arrhythmic risk [86,87]. Although hERG potassium channels play the central role in drug-induced arrhythmias, the contribution of other CM ion channels in the arrhythmogenic effect of some drugs is doubtless [88,89]. The combination of high-throughput optical measurements with HiPSC derived CMs allows evaluation of the overall effects of candidate molecules on heart electrophysiology.

The all-optical Optopatch approach was utilized in one of the early attempts to develop such a cardiotoxicity assay [90]. In this screening concept two populations of hiPSC derived CMs were cultured together in a mixture: one subset expressed CheRiff actuator [74], and the other expressed the fusion protein CaViar (Ca^2+^ and Voltage indicator [91]), consisting of the voltage sensor QuasAr2 [74] and the Ca^2+^ indicator GCaMP6f [92]. CheRiff-expressing cells were used for pacing the entire syncytium of cardiomyocytes by blue light pulses, while CaViar allowed for simultaneous recording of the resulting membrane potential and Ca^2+^ fluctuations in the other CM population. Although not tested in real screening, Cardiac Optopatch has shown good performance in testing compounds with known mechanisms. It successfully demonstrated the effects of drugs on action potential waveforms and Ca^2+^ dynamics in spontaneously beating cultures and cultures paced at different frequencies. Moreover, the screening platform was proven to be suitable for studying long-term drug effects, which may allow it to be used in delayed drug cardiotoxicity assays.

The alternative mixed electrical-optical approach employing a combination of field stimulation of hiPSC derived CMs with GEVI recording has also demonstrated its applicability for cardiac drug evaluations. In the absence of an optogenetic actuator the use of red-shifted voltage indicators becomes non-obligatory, and non-opsin based GEVIs were employed. VSFP-CR, a FRET-based voltage sensor consisting of the voltage sensing domain of a potassium channel and a GFP/RFP FRET pair [93,94] was used for cardiomyocyte subtype-specific AP imaging [95]. Placing the sensor under the control of lineage-specific CM promoters made it possible to detect and measure the changes in AP duration and the occurrence of early afterdepolarizations caused by deleterious mutation or induced by drugs in patient-specific hiPSC-derived ventricular-, atrial-, or nodal-like cardiomyocytes. ArcLight A242 [96], a variant of *Ciona intestinalis* voltage-sensing phosphatase (CiVSP)-based sensors containing fluorescent protein super ecliptic pHluorin, was utilized in another series of experiments [97,98,99]. ArcLight-expressing hiPSC-derived cardiac cell sheets (hiPSC-CCSs) were used for optically mapping the electrical activity in a two-dimensional cardiac tissue model during different experimental conditions, including electrically- and drug-induced arrhythmias and arrhythmia-preventing interventions [98].

One of the concerns related to the use of genetically encoded indicators for HiPSC-based drug screening is possible gene disruption or gene expression changes caused by multiple and random genome integrations of the lentiviral vector used for GEVI delivery. This concern was addressed by using the CRISPR/Cas9 system for ArcLight gene integration into the AAVS1 safe harbor locus of HiPS cells [99]. The resulting ArcLight-expressing HiPSC and HiPSC-CMs line demonstrated stable long-term expression of ArcLight, enabling repeated electrophysiological recordings as early as 21 days and for at least 162 days postdifferentiation. Surprisingly, the differentiated ArcLight-CMs appeared to be brighter than ArcLight-HiPSC before differentiation. Such targeted CRISPR/Cas9 based gene integration seems more appropriate than viral delivery and other transfection methods for GEI-based assays.

Thus, the GEVI-based optical electrophysiology approach to drug screening has proved its suitability in several proof-of-concept studies. Nevertheless, to the best of our knowledge it is still not used in real high-throughput assays. In addition to the relative novelty of the method the reason for that seems to be the weaknesses of the existing GEVIs, mainly the low brightness of opsin-based sensors, and the slow kinetics of non-opsin based sensors [75]. It is no coincidence that in the latest published evaluation of the Optopatch-based drug screening approach, QuasAr2 was replaced with the chemical voltage sensitive dye BeRST1, that requires a more than 20-fold lower intensity of red light illumination [84]. However, although voltage sensitive dyes do indeed provide a good alternative to GEVIs, and in some cases outperform them, genetically encoded indictors still have many advantages, the most important of which are the absence of phototoxicity, consistent expression with no need for repeated loading, and easy targeting to specific cell types using cell specific promoters. The development of brighter and faster genetically encoded voltage indicators compatible with current high throughput imaging approaches will help enable early-stage testing of drug candidates for their possible interactions with VGCs, and prevent potentially cardiotoxic molecules from entering preclinical and clinical trials, decreasing drug development cost, and improving speed and efficiency.

## 5. Fluorescent Sensors Detecting Drug Toxicity

### 5.1. Proteome Stress Inductors Screening

Proteome stress includes protein misfolding and aggregation, and this can be caused by drug treatment. These processes may precede the development of cellular pathology and death, and it is important to be able to determine them. The protein-based fluorogenic proteome stress sensor AgHalo is able to detect soluble aggregates formed in the early stages of cell stress as well as insoluble aggregates formed later. The sensor is based on the protein tag (HaloTag) which is a modified haloalkane dehalogenase [100]. Haloalkane dehalogenases remove halides from aliphatic hydrocarbons by a nucleophilic displacement mechanism. This protein is modified for covalent binding to synthetic ligands (P1 ligands). The ligands comprise a chloroalkane linker that takes part in the formation of covalent bonds by nucleophilic displacement of the terminal chloride with aspartate located in a deep pocket of the protein part of the sensor. In the case of AgHalo the P1 ligand contains the solvatochromic fluorophore sulfonylbenzoxadiazole (SBD) that has polarity-dependent fluorogenicity providing specificity of unfolded sensor fluorescence and minimal cellular background fluorescence, and the extended sarcosine linker minimized undesired fluorophore interactions with the protein part of the sensor in the folded condition. AgHalo harbors a K73T mutation and exhibits reduced stability (ΔGfolding = −2.0 kcal/mol) relative to wild-type Halo (ΔGfolding = −5.6 kcal/mol). That is why the sensor unfolds under proteome stress and is sensitive to aggregation, that initiates fluorescence. AgHalo works by an aggregation-specific turn-on fluorescence mechanism. Temperature-induced aggregation of AgHalo-P1 conjugate results in a 10-fold increase of 545 nm fluorescence [101].

Five anti-cancer drugs which according to the LDH test did not exhibit cytotoxicity were tested on Hek293T cells using AgHalo. Live cell imaging data were confirmed by fractionation experiments and confocal fluorescence microscopic imaging. Drug-induced proteome stress caused AgHaloP1 conjugate fluorescence. Fluorescence intensity decreased through the sequence: Nilotinib, Pemetrexed, Imatinib, Thalidomide, and Carboplatin. Nilotinib (50 µM, 24 h) showed the highest fluorescence increase due to aggregation of the AgHaloP1 conjugate whereas Carboplatin at the same concentration and time exposure had a minimal effect.

### 5.2. Mitochondrial Toxicants Screening

Mitochondrial toxicants are compounds that cause a decrease in the number of mitochondria within a cell, and/or decrease the ability of mitochondria to perform normal functions including producing adenosine triphosphate and maintaining cellular homeostasis. Mitochondrial dysfunction can lead to apoptosis, necrosis, altered metabolism, muscle weakness, neurodegeneration, decreased organ function, and eventually disease or death of the whole organism. It is known that mitochondrial poisons and ETC inhibitors (rotenone a complex I inhibitor, antimycin a complex III inhibitor, azide a cytochrome c inhibitor, and oligomycin, an ATPase blocker) induce activation of glycolysis and lead to an increase in cytosolic lactate [102,103]. Based on these observations intra-cellular lactate levels can be used as a wide specificity readout of mitochondrial dysfunction.

The MitoToxy Assay was developed by Contreras-Baeza et al. for in vivo HTS of lactate based on the genetically encoded FRET indicator Laconic. Laconic (LACtate Optical Nano Indicator from CECs) contains the full-length transcription regulator LldR from *E. coli* carrying two modules, a lactate binding/regulatory domain and a DNA-binding domain. LldR connected with a FRET pair in which mTFP acts as donor and Venus as acceptor. Lactate binding results in an acceptor-to-donor emission ratio decrease. The sensor was capable of quantifying lactate levels in the range between 1 mM and 10 mM, which corresponds to physiological concentrations. The MDA-MB-231 breast adenocarcinoma cell line grown in galactose rich media; was used as a cell platform for HTS of lactate in the MitoToxy Assay since it had the lowest Warburg index (glycolytic/oxidative metabolism quotient) among the cells tested and was as oxidative as a primary astrocyte culture with a known high oxidative phenotype. To increase the amplitude of toxicity-induced lactate accumulation and decrease data variation lactate efflux was blocked by the MCT inhibitor pCMBS with broad specificity, the blockade included MCT4, which is the main monocarboxylate transporter present in MDA-MB-231 cells [104]. The high mitochondrial activity of MDA-MB-231 cells cultured in the described conditions makes cells sensitive to the minimal concentration of known mito toxicants. Suitability for high-throughput screening applications was evaluated in a Z′-factor > 0.5 and inter-assay coefficient of variation of <20%. Thirteen compounds were chosen from a variety of therapeutic classes to perform a pilot screening assay using MitoTox Reporter cells and a standard multiplate reader. The pilot screening detected time and dose-dependent toxicity of thiazolidinediones such as ciglitazone, troglitazone, rosiglitazone and the high toxicity of the anti-cancer drug camptothecin at 5 μM, whereas the mitochondrial effects of the antihistamine terfenadine and the anti-cancer drug flutamide were detected at 10 μM. As a reference, the maximum concentration of these drugs in plasma (Cmax) lies in the range of 3 to 10 μM. It is worth noting one false negative result. Nilutamide did not produce ΔR% changes at any of the concentrations tested, in spite of its known toxicity profile. A possible explanation for this behavior is that the drug stops pyruvate consumption, but without secondary glycolytic activation, so the amount of lactate is not enough to produce saturation of the sensor.

Redox sensitive GFP with mitochondrial localization (mt-roGFP2) was chosen by Chandrasekharan et al. as a fluorescent sensor for the creation of a HTS platform detecting drug-induced mitochondrial damage [105,106]. The sensor roGFP2 has two excitation maxima at about 400 and 475–490 nm and a fluorescence maximum at about 520 nm. Ratiometric variation of fluorescence excitation of roGFP is caused by an increase in the chromophore protonation state upon oxidation. For roGFP2, oxidation decreases the 400-nm excitation peak while proportionally increasing the 475-nm peak. Creation of the mt-roGFP2 HTS of drug-induced mitochondrial damage was possible due to the correlation between the mt-roGFP2 detectable increase in reactive oxygen species (ROS) production in U2OS osteosarcoma cancer cells and drug-induced mitochondrial permeabilization detected by apoptotic cytochrome c-EGFP release. Activation of Bax-EGFP and its translocation into mitochondria, an early apoptotic event, also correlated with drug-induced mitochondrial damage. Moreover, drug-induced ROS production correlated with increased Annexin-V cell positivity and mitochondrial membrane potential loss visualized by loss of TMRM staining. The authors showed that cisplatin induced an increase in the mt-roGFP2 ratio that started prior to mitochondrial permeabilization, which was detected by release of Smac-mCherry from the intermembrane space and reflected ROS production. Thus, ROS detection was chosen as a criterion for assessing the early stages of mitochondrial damage. Cells stably expressing mt-roGFP and H2B-mCherry were developed for real-time imaging of mitochondrial oxidation and were the basis of the development of the image-based assay for screening cytotoxic compounds in a high-throughput manner. Fast screening of 96-well optical bottom plates could be performed within 15 min in a 2 × 2 montage with a BD Pathway™ 435 Bioimager. Red fluorescence of nucleus allowed complete and accurate automatic perinuclear region segmentation followed by mt-roGFP ratio calculation. The calculated Z factor for the OVCAR8 cells with nuclear H2B-mCherry was 0.71 as a result of the accurate segmentation. A set of known drugs with different structures and actions were tested in the mt-roGFP HTS assay and relative mitochondrial damage exerted by each compound was evident by FACS measurement of the mt-roGFP ratio, TMRM intensity, and Annexin V staining. Only nigericin, which is a known inducer of inflammatory caspase1 mediated cell death, at 1–2 μM and with 24 h incubation did not induce ROS production among all tested compounds. The imaging-based HTS of mitochondrial ROS production described here may have promising applications in drug screening and mitochondrial toxicity assessment.

## 6. Anti-Parasitic Drug Screening

Despite the efforts of the World Health Organization, malaria remains the scourge of Africa. In 2018, an estimated 228 million cases of malaria occurred worldwide. The African Region accounts for the majority of cases (213 million or 93%). Thousands of people die from malaria every year. In 2018, there were an estimated 405,000 deaths from malaria globally, with children under 5 accounting for 67% (272,000) of deaths [107]. Many antimalarial drugs are toxic and some develop drug resistance. Therefore, the search and creation of antimalarial drugs remains an extremely important task in research and pharmacology.

Glucose is the primary source of energy for blood-stage parasites for biomass production and ATP synthesis. The hexose/glucose transporter (PfHT) of the prevalent malaria parasite *Plasmodium falciparum* is well-known. It has been demonstrated that PfHT is essential for parasite survival, and it has been validated as an antimalarial target [108,109]. Using the human embryonic kidney cell line HEK293, and knocking down the primary endogenous transporter isoform GLUT1 using shRNA, Kraft et al. have created a cell line in which glucose is transported almost entirely by the PfHT transporter, which is stably expressed. They combined this cell line with an intracellular glucose fluorescent FRET sensor protein as a readout. They used the glucose FRET sensor FLII12Pglu-700 μδ6 (FLIP) which contains a glucose-binding central domain terminated with CFP and YFP. Binding of glucose causes an increase in FRET as a result of conformational changes in the sensor that bring the donor and acceptor closer together [110]. This FRET sensor mediated cell-based glucose assay forms the basis of highly specific HTS screenings of PfHT inhibitors in plate reader format (Z′ factor of > 0.8) [111,112]. The specificity of hits was confirmed through a subsequent counterscreen using the same HEK293-FLIP cell line overexpressing one of the class I transporters and knocking down the others to reduce background glucose uptake. The counterscreen allowed selection of hits with unique inhibitory properties specific only to PfHT. A counterscreen of five PfHT inhibitory hits selected from a library of 400 compounds known to inhibit erythrocytic development of *P. falciparum* (Medicines for Malaria Venture (MMV) Malaria Box) were made against the human orthologues GLUT1, -2, -3, and -4. Compound MMV009085, the most potent hit, with an IC50 of 2.6 μM for PfHT-mediated glucose uptake, showed significantly less inhibition of the human GLUTs than for PfHT [111]. Hit confirmation was ascertained by determining the IC50 for glucose uptake into isolated *P. falciparum* parasites from blood culture using radiolabeled d-glucose. The same HTS of the Maybridge HitFinder library of 14,399 compounds revealed 6 hits, confirmed after radiolabeled 2-deoxy-glucose (2-DG) uptake inhibition in PfHT-overexpressing HEK293 cells. Compound WU-1 (3-(2,6-dichlorophenyl)-5-methyl-*N*-[2-(4-methylbenzenesulfonyl)ethyl]-1,2-oxazole-4-carboxamide) exhibited potency for PfHT inhibition in the low micromolar range, efficacy in inhibiting parasite growth and excellent selectivity against the human GLUT isoforms (class I (GLUTs1–4), class II (GLUT8) and class III (GLUT5) when compared to the other confirmed hits identified in the screen [112].

The above described FRET sensor mediated cell-based screening system is easily adaptable to specific glucose transport through selectively expressed glucose transporter isoforms. This opens up opportunities for HTS for glucose transporter inhibitors, common targets in anticancer therapy.

The kinetoplastid parasite *Trypanosoma brucei*, transmitted by tsetse flies, causes Human African trypanosomiasis (sleeping sickness) in sub-Saharan Africa. Obsolete and toxic drugs are the common treatments for trypanosomiasis [113]. Glucose metabolism in kinetoplastid parasites is spatially localized in specialized peroxisome-like organelles known as glycosomes, were ATP production occurs from glucose metabolism while in the mammalian host. The parasite-specific glucose transporters THT1 and THT2 have unique biochemical characteristics that differentiate them from mammalian glucose transporter homologs. Point of action glycolytic path inhibitors for specific drug development may be located in THT1 or THT2.

An assay for monitoring changes in cytosolic and glycosomal glucose levels in living *T. brucei* using a fluorescent protein biosensor (FlII12PGlu-600 μ) in combination with flow cytometry was developed by Voyton et al. and became the basis for HTS of trypanosome glucose transporter inhibitors [114]. A decrease in the the Venus/CFP emission ratio of the glucose sensor FLIPglu-600 µ on the addition of glucose was the readout for the assay (less energy is transferred by FRET to Venus and the CFP emission levels increase) [115]. Glycosome targeting of the FRET sensor by fusion with a peroxisomal targeting signal and assessment of the efficiency of bloodstream form parasite killing activity of the selected inhibitors prevent the problem of poor cell permeability, off-target effects, and general cytotoxicity. There were two active compounds among the 400 compounds of the small molecule Pathogen Box library provided by Medicines for Malaria Venture (MMV) that contains 70 well-characterized anti-kinetoplastid drugs as well as reference anti-trypanosomal compounds including suramin and pentamidine. Compound MMV085210 inhibited both cytosolic and glycosomal glucose uptake, but due to poor solubility and low concentration did not appreciably impact bloodstream form (BSF) parasite viability. Another hit (MMV272144) provided only inhibition of glycosomal glucose and did not affect cytosolic levels. In addition to inhibiting glucose uptake with relatively high potency (EC50 = 700 nM), the compound also showed modest bloodstream form parasite killing activity (41% killing) and may be attractive as a potential anti-trypanosome drug.

## 7. Ca^2+^-Signaling Modulator Screening

Ca^2+^, being a universal participant in various cellular processes, is an important target for high-throughput screening. Cell-based Ca^2+^ assays are widespread in pharmaceutical studies to examine functional responses of membrane receptors and ion channels. Since alterations in Ca^2+^ homeostasis occur in a number of pathological processes (neurological disorders, cardiovascular disease, cancer), Ca^2+^ imaging is used for drug development, creation and screening [116,117].

Nowadays Genetically Encoded Calcium Indicators (GECI) are indispensable tools for Ca^2+^ imaging predominantly in neuroscience. Though, their field of use is extending to other areas, including for HTS. Using primarily GFP color variants and a series of red and photoconvertible fluorescent proteins in combination with Ca^2+^-sensing domains a wide panel of probes have been developed in recent years [118,119,120]. New generation Ca^2+^ indicators are characterized by improved sensitivity and dynamic range which make them comparable with widely used standard synthetic dyes. GECI types, structure, and physicochemical properties are extensively reviewed [121,122,123,124]. Compared to the widespread synthetic dyes used for Ca^2+^ measurement, GECI usage makes HTS cheaper (no high cost dyes), faster (no time-consuming dye-loading step), and reproducible (no great variability between assays due to the need to use freshly prepared dye). Moreover, GECI usage overcomes the problem of the limited detection time window inherent in synthetic dyes and allows long-term monitoring of intracellular Ca^2+^ dynamics.

Wu et al. developed a cell-based assay using a 293-F clonal cell line stably-expressing the green single-wavelength GECI GCaMP6s and compared its performance with fluo-4 by testing the potencies of pharmacological agents targeting TRPV1 and muscarinic receptors. The well-known potential cytotoxicity associated with long-term expression of GECIs may trigger negative selection pressure on a clonal cell line and can cause loss of expression of the gene of interest when using standard plasmids in which the antibiotic selection marker is physically separated from the GECI. Connection of the GCaMP6s and Blasticidin-S resistance genes via the self-cleaving porcine teschovirus 2A sequence prevented this problem and allowed for near-stoichiometric expression of two separate proteins driven by a single promoter. In comparative experiments the genetically-encoded fluorescent sensor and synthetic dye showed similar dynamic range (the ratio of the responses of the negative controls to the fully-inhibited controls), close effective dynamic range (saturated fluorescence divided by the fully-inhibited response), and reproducibility. The assay quality parameter z’ reflecting the reproducibility of the control responses exceeded 0.5 for both Ca^2+^ indicators for each receptor type [125]. The potencies of a panel of pharmacological agents targeting TRPV1 and the muscarinic receptor were comparable for the two indicators. Thus, Wu et al. demonstrated a legitimate use of GCaMP6s cell-based assay in high-throughput pharmacology screening.

A study by Cai et al. demonstrated the flexibility of using GECIs which can be modified with molecular biology tools to optimize an indicator for a specific task. They developed a novel indicator dCys-GCaMP by introducing a cysteine pair to the neighboring β-sheets of the barrel structure of the cpEGFP domain of the chromophore in GCaMP3 to decrease background fluorescence from dead and damaged cells. Disulfide bond formation between cysteine pairs in damaged and dead cells disrupt the barrel-like structure of the fluorophore and reduces fluorescence even with Ca^2+^ binding. The background fluorescence in HEK 293 cells transfected with GCaMP3 was approximately 30% higher than in experiments with dCys-GCaMP. dCys-GCaMP was applied to a Ca^2+^ flux assay for NMDA receptor types NR1/NR2A and NR1/NR2B and the α1A adrenergic receptor (α 1-AR), all of which considered important drug targets. In response to agonist stimulation of the NMDA receptors, the indicator showed a strong fluorescence signal, a high signal-to-noise ratio (S/N) (20 and 14 for NR1/NR2A and NR1/NR2B, respectively) and excellent assay sensitivity for both receptor types with high reproducibility (Z′ > 0.7). Ca^2+^ flux assays of NMDA receptors treated with an agonist, antagonist, channel blocker, and allosteric modulator, performed using both fluo-4 or dCys-GCaMP demonstrated close results. The performance of dCys-GCaMP in a library screening of 66 known NR2B inhibitors also demonstrated the robustness of the indicator and this was confirmed with fluo-4. In experiments with cells expressing α 1-AR, both indicators also showed comparable results [126]. Thus, the usefulness of GECIs in drug screening searches was confirmed once again.

One of the most striking examples of using GECI for HTS concerns drug development studies for Alzheimer’s disease (AD). Intracellular Ca^2+^ homeostasis dysregulations occur in early AD and are regarded as one of the causes of the serious brain impairments at later stages. The possibility of normalizing Ca^2+^ levels in the endoplasmic reticulum store, is therefore a potential target for the discovery of a new effective therapy [127]. For this purpose, a fully automated high-throughput cell-based Ca^2+^ assay utilizing the FRET-based indicator Yellow Cameleon 3.6 was developed for compound screening. HTS was performed on a HEK293 cell line stably expressing a Presenilin 1 (PS1) gene mutant variant linked with early onset familial AD (FAD) that mediates ER Ca^2+^ homeostasis disruption. PS1 mutant expressing cell lines demonstrated augmented muscarinic receptor agonist carbachol (CCh)-evoked Ca^2+^ release (three-fold higher than in a wild type PS1 cell line) and were used as the target to screen for compounds that can reverse exaggerated Ca^2+^ release towards physiological levels. The assay used the 384-well optical bottom plate format, with single-cell-based microscopic detection of the intracellular Ca^2+^ level in combination with automated image analysis enabling the detection of even slight changes in Ca^2+^ levels which cannot be achieved by conventional single-well-based Ca^2+^ measurement screening technologies. The average Z′-factor for the ten randomly selected plates that were screened exceeded 0.8 [128]. The assay was used to screen a library of 20,000 compounds of which 53 active compounds of 4 lead structures were identified. Those structures belonged to following compound classes: Thiazolidine, Phenothiazine, Imidazole and Benzhydrilpiperidinamine. The majority of identified active compounds showed no toxicity and HEK293 cells treated with 10 µM of the compounds for 24 h remained viable. It is important that the activity of the identified hits is not only specific to the FAD-PS1 mutation PS1-M146L used in the primary screen, but is present across other PS1 mutations as well. The analysis also confirmed the previously known effectiveness of the Ca^2+^ antagonist drug Bepridil against exaggerated ER Ca^2+^ release. The team used a similar approach to study the effect of tetrahydrocarbazoles on Ca^2+^ homeostasis as potentially effective drug candidates for AD [129].

The use of human induced pluripotent (iPSC) stem cells derived from patients is a promising direction in the development of drug screening. For neuropsychiatric disorders’ models, iPSCs can be applied to derive neural circuits. Fantuzzo et al. developed a proof-of-concept platform that can be applied to initial drug screening in distinct populations of induced neural cells (iNCs) in a high-throughput fashion. They created a 96-well device where excitatory and inhibitory iNCs were seeded in two distinct chambers in each well connected by microchannels to model simple neural circuits. All cells were preliminarily transduced with GCaMP6f to visualize circuit activity and connectivity. Excitatory cells were additionally transduced with a designer receptor exclusively activated by designer drug (DREADD) based on human muscarinic receptor type 3. To demonstrate the formation of functional circuits and HTS applicability, cells expressing the designer hM3Dq receptor were stimulated with the designer ligand clozapine-*N*-oxide (CNO) the signaling Ca^2+^ mobilization and post-synaptic response were measured. Increasing agonist concentration resulted in increasing cell activity in both chambers, indicating the presence of excitatory inputs through the chambers’ microchannels. The proposed platform provides a powerful means for automated HTS of therapeutic reagents in established neurocircuits carrying the genetic context of a patient and may be used for testing personalized drug therapies as well [130].

As Ca^2+^ plays a crucial role in muscle contraction, Ca^2+^ imaging might serve for investigation of Ca^2+^ alterations in cardiovascular disease. The cardiac isoform of the sarco/endoplasmic reticulum Ca^2+^ ATPase (SERCA2a) that regulates Ca^2+^ reuptake into the SR is an important therapeutic target for heart-failure drugs. Several studies have implemented GECIs based on SERCA2a fused with FPs to assess Ca^2+^ signals detected by FRET in HTS platforms for drug discovery. FRET monitors the structural status of SERCA, which is affected by the binding of small molecules. Schaaf et al. proposed a HTS strategy using an intramolecular FRET indicator composed of GFP and RFP fused to SERCA2a’s N-terminus and nucleotide-binding domain correspondingly [131]. An improved red-shifted version of the indicator utilizing the orange florescent donor mCyRFP1 and the far-red acceptor mMaroon1 as a FRET pair for a HTS lifetime-based assay was introduced later [132]. The donor’s fluorescence lifetime (FLT) measurement contributed to an increase in the precision of detection of SERCA2a structural changes caused by interactions with small molecules. The longer fluorescence lifetime of the red-shifted donor improved the dynamic range of the sensor. As a result, the Z′-factor increased from 0.62 (GFP/RFP) to 0.75 (mCyRFP1/ mMaroon1) reflecting an improvement in the quality of the assay.

**Figure 4:**
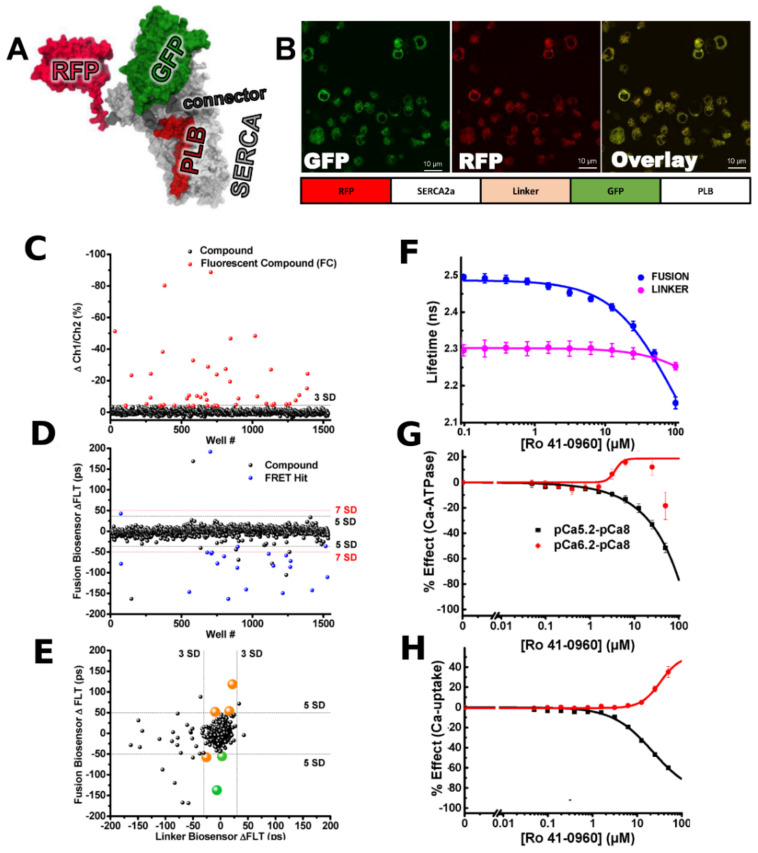
(**A**) Molecular model of concatenated sarcoplasmic reticulum Ca-pump (SERCA2a)-phospholamban (PLB) biosensor, based on crystal structures for SERCA2a-PLB (4KYT), red fluorescent proteins (RFP) (3M22), and GFP (1GFL). (**B**) Confocal microscopy of HEK293-6E suspension stable clone expressing the fusion biosensor at the ER membrane. (**C**–**E**) Library of pharmacologically active compound (LOPAC) screen results: (**C**) Ch1/Ch2 intensity ratio flags 27 fluorescent compounds (FC; red). (**D**) Compounds that change the SERCA2a-PLB fusion biosensor FLT by ≥ 5 SD were selected as Hits (blue). FCs were flagged and excluded from Hits. Two Hit thresholds are shown, 5 SD and 7 SD (black and red dotted lines, respectively. (**E**) Hits were further triaged by counter screening using the cell line expressing the GFP-RFP linker biosensor. Two reproducible Hits (thapsigargin and Ro 41-0960, shown in green) passed the linker biosensor test by changing its FLT by < 3 SD. Both compounds are known SERCA2a effectors. The remaining Hits are shown in orange. (**F**–**H**) Concentration-response curve (CRC) analysis of LOPAC FRET Hit Ro 41-0960. (**F**) FLT CRCs, using the fusion and linker biosensors, as indicated. (**G**) SERCA2a Ca-ATPase assays CRCs at [Ca^2+^] corresponding to maximal activity (pCa 5.2, black), intermediate (pCa 6.2, red). Basal activity (pCa8) was subtracted from each. (**H**) SERCA2a Ca^2+^-uptake monitored at pCa 6.2 (red) and 5.2 (black) using the Ca-sensitive dye Fluo-4 fluorescence. Each experiment was performed in duplicate (Ca-ATPase) or triplicate (Ca-uptake); error bars indicate SEM (n = 2 or 3). Curves generated using Hill fit. The figures were taken from Schaaf et al., 2020 with minor changes [133].

To discover compounds that raise SERCA activity, an intramolecular FRET sensor based on complex of SERCA2a and its regulator phospholamban (PLB) was created. PLB regulates cardiac contraction by inhibition of the sarco/endoplasmic reticulum Ca^2+^ ATPase SERCA. Unlike the previous indicators, where binding of a substance to SERCA leads to conformational changes, this sensor allows identification of compounds that disrupt SERCA’s inhibitory complex with PLB. The sensor was made by fusing RFP-SERCA2a and GFP-PLB through a flexible 47-residue peptide linker on a single-polypeptide chain (Figure 4A). Monoclonal HEK293-6E cell lines stably expressing the RFP-SERCA2a-LINKER-GFP-PLB biosensor were generated and the retention of functional activity and normal Ca^2+^-dependent inhibition by PLB were shown (Figure 4B). Dual-wavelength fluorescence lifetime (FLT) detection in the plate reader format was performed in living cells for HTS of the library of pharmacologically active compounds (LOPAC) with the SERCA2a-PLB fusion biosensor. At first, false hits caused by compound fluorescence were effectively filtered out by simultaneous two-channel donor emission detection (Figure 4C). Donor emission was split into two channels: Ch1—peak of the GFP emission spectrum (517 nm), and Ch2—43% of the GFP emission spectrum (535 nm). Compounds that changed FRET ratio did not affect the Ch2/Ch1 ratio significantly. In contrast, fluorescent compounds dramatically increased the Ch2/Ch1 ratio (>3SD of the DMSO control average). This proves that interference from fluorescence of the test compound did occur. At the second step of the screening FRET hits were assessed using ∆FLT in the primary detection channel at 517 nm. Hits were selected based on a 5 or 7 SD threshold followed by counter-screening against a mock biosensor construct consisting of GFP tethered to RFP via a 32-residue flexible linker (Figure 4D,E). This mock biosensor reveled compounds that interact with fluorescence proteins and change ∆FLT in false positive manner. Ro 41-0960 (catechol-*O*-methyltransferase (COMT) inhibitor) was identified as a small-molecule that increases SERCA2a’s activity at a physiologically relevant Ca^2+^ concentration while at a high level of Ca^2+^ Ro 41-0960 inhibited the Ca-transport activity of SERCA2a (Figure 4F–H). The FRET increase caused by Ro 41-0960 indicates that it acts by changing the conformation of the SERCA2a-PLB complex rather than by dissociating PLB from SERCA2a [133].

## 8. Animal Models for Drug Screening Using Genetically Encoded Sensors—Looking Towards the Future

### 8.1. Animal Models for Drug Screening

Despite the progress in drug research, the success rate from phase I through to launch remains extremely low resulting in an average cost for bringing a new drug to the market of around $2.5 billion [134,135]. One of the reasons for this is insufficient attention to relevant preclinical disease models in the early stages of research. Immortalized cell lines often poorly represent human diseases. One effective way to overcome this issue is to use primary cultures of hiPSC (human induced pluripotent stem cells) derived cells [136]. The main disadvantage of using cell cultures is that they can’t form the complex cellular environment of the tissue, which can be critically important for drug efficiency and delivery testing. For that purpose, small organoids or complex organ-on-chip systems can be used [137]. Another effective approach is the use of whole animals for drug screening, which provides the physiological context of the tissue, organ and organism.

Several animal species have been adapted for HTS. Despite mice and higher animals being well studied and widely used in drug research, they do not scale to HTS pannels. While the rapid development of robotics could potentially overcome present limitations, for now, all animals used in HTS possess certain qualities—that is relatively small size, high fertility, rapid development, and inexpensive maintenance. Transparent worm Caenorhabditis elegans is frequently used in HTS, because of its microscopic size and fast life cycle, which makes it extremely cost-effective. Despite the positive aspects, evolutionary distance from humans and anatomical simplicity significantly limits its use [138]. Another invertebrate animal used in HTS screening is Drosophila melanogaster fly. More complex in structure, it retains the qualities required for HTS, and has a larger number of orthologs for human genes, associated with diseases [138]. The closest human relatives used in HTS screening are vertebrate Oryzias latipes (medaka) and Danio rerio (zebrafish) fishes [138,139]. Among them zebrafish has garnered the most attention for several reasons. These are a large set of basic research on the model, the fact it is a vertebrate, its low-cost maintenance, small size, external and rapid development, high fecundity, and the transparency of larvae, which can be enhanced using PTU (1-phenyl 2-thiourea) melanization inhibitor or zebrafish lines that remain transparent as adults [139,140,141,142]. While a distant relative of humans, 71.4 percent of human genes are present in zebrafish, and about 80 percent of the genes known to be associated with human diseases have some counterpart in zebrafish [143]. Though 47 percent of the orthologous genes have only one ortholog for one human gene, the remaining 53 have two or more orthologs for one human gene, which must be considered when modelling diseases [143]. The zebrafish genome is well annotated and can be browsed with the GRC or ZFIN databases.

### 8.2. CRISPR Technology in Human Diseases Modeling

Recent advances in CRISPR technology have greatly facilitated the creation of accurate zebrafish models of genetic human diseases. The first paper on CRISPR genome editing in zebrafish was published in January 2013 [144], the same month all original papers on the effectiveness of CRISPR for genome engineering were published [145,146,147]. The protocol for CRISPR editing in the study was simple and effective—sgRNA for the targeted gene and mRNA encoding spCas9 protein were injected into one-cell-stage zebrafish embryos. This system has shown successful knock-out for more than 80 percent of the sites tested (9 out of 11) [144]. The next paper reported the possibility of biallelic knock-outs and successful knock-in events in genes of interest upon addition of ssODN (single-stranded DNA oligonucleotide) donor templates to the system [148]. In a subsequent paper it was shown that with CRISPR technology it’s possible to target multiple genomic loci in zebrafish (5 in the performed study) [149], which is especially important for the generation of genetic patterns for different disease models. Further improvement of CRISPR methods showed an 85 percent knock-out success rate (138 of 162 sites) and a germline transmission rate of about 28 percent (2097 of 7525 embryos from 1080 founders) [150]. Therefore, the generation of stable zebrafish models with CRISPR is straightforward and can be achieved in F1 or in F2 animals. Germline screening can be performed using the standard set of molecular biology methods such as Sanger sequencing, allele-specific PCR, restriction enzyme digestion and heteroduplex mobility assay [151,152].

Different versions of CRISPR technology for zebrafish with improved characteristics were designed. Among them, those with increased specificity or efficiency are of most interest. CRSIPR systems with PAM sequences different from the spCas9 NGG sequence can be viewed elsewhere [153]. Delivery of Cas9 and sgRNA in the form of a pre-assembled ribonucleoprotein complex noticeably increases the rate of mutagenesis upon injection in some cases [150]. Zebrafish mutants generated with this method are commonly referred to as crispants (analogous to morpholino generated morpants). Another approach uses simultaneous injection of different sgRNAs targeting the same allele, which increases the possibility of gene knock-out in a predictable manner [154,155]. Many possible structures of the donor DNA template are available for knock-in editing. It has been shown that in zebrafish ssODN donors cause mutations on the ends of the insertion site, disrupting desired sequences with high frequency, but for small region editing the ssODN template may be sufficient enough [156,157]. The most precise and ready to use templates are small donor plasmids with homology arms of around 1000 bp flanking the insertion sequence [158]. Editing efficiency can be further enhanced by in vivo linearization of donor plasmids [158,159].

Integration of a CRISPR cassette under the control of a tissue specific promoter with the classical Tol2 technique in the zebrafish genome provides tissue-specific knock-out of selected genes [160,161]. Additionally, use of GAL4 tissue specific zebrafish lines allows the use of the robust UAS promoter for Cas9 protein thereby increasing the effectiveness of the knock-out [162]. More than that, incorporation of Cre recombinase into the CRISPR cassette allows permanent labeling of Cas9-expressing cells in combination with zebrafish lines carrying a reporter fluorescent cassette activated by Cre recombinase [162]. Zebrafish lines carrying Cre recombinase under the control of a tissue specific promoter can be used for the creation of tissue-specific gene knock-out with a Cas9 cassette containing a STOP sequence surrounded by Cre target sites [161]. In this case, a gene trap between loxP sites is cut out in a tissue of interest, allowing expression of Cas9. In combination with heat shock or drug-inducible promoter this method provides an opportunity for temporal control of editing. Photoactivated Cas9 or gRNA molecules provide light-mediated knockout with high spatiotemporal resolution [163]. With the CRISPR knock-in approach it is possible to generate loxP sites at the ends of genes of interest and use a zebrafish line with Cre recombinase under the control of a desired promoter for conditional knock-out [164].

Another editing approach is single “base editing”. The CRISPR instrument BE3, consisting of nickase Cas9 protein fused with rAPOBEC1 cytidine deaminase and UGI (uracil DNA glycosylase inhibitor) provides the ability for precise C → T conversion within a window of approximately five nucleotides [165]. This technology makes it possible to efficiently produce point mutations related to human diseases in zebrafish [166,167]. Another Cas9 modification ABE, consisting of nickase Cas9 protein fused with a mutated version of ecTadA adenine deaminase provides A → G conversion within a window of approximately three nucleotides [168]. With slight modifications this system has been successfully used in zebrafish [169]. The CRISPR “base editing” toolkit is constantly being improved, creating new possibilities for editing [170,171].

Recently, a novel CRISPR strategy for genome editing without the generation of a double-strand DNA break was developed. This method utilizes Cas9 nickase fused with mutated M-MLV (moloney murine leukaemia virus), reverses transcriptase and pegRNA as the guide and donor template, and provides highly efficient editing of small regions in the genome [172]. The method is yet to be tested in zebrafish, but could provide interesting application prospects in the case of successful translation.

Despite the novelty of CRISPR technology, it has successfully been used to generate a significant number of genetically relevant models of human diseases in zebrafish. Partly because the ease of obtaining zebrafish knock-out mutants has led to widespread validation of disease-associated genes identified by GWAS (genome-wide association studies) using zebrafish. Human diseases recreated with zebrafish include retinal diseases, nervous system diseases, cardiovascular diseases, immunological diseases, cancer and many others [173,174].

### 8.3. Fluorescent Sensors in Zebrafish Research and Drug Screening

Most drug screening on zebrafish uses phenotypic analysis, behavioral assays, fluorescent proteins, or fluorescent dyes as reporters with genetically encoded fluorescent sensors being overlooked despite their qualities [138]. In spite of the low representation in the HTS field, a number of fluorescent sensors have been successfully tested and applied for basic research in zebrafish (Table 1).

Danio rerio, despite being a relatively new animal model, has nevertheless been successfully used in the first steps of drug screening that directly led to subsequent human trials. The dmPGE2 molecule for transplantation therapy was discovered during zebrafish screening for new hematopoietic stem cell formation and homeostasis modulators [175,176]. Zebrafish screening for melanoma growth inhibitors has led to the discovery of qualities of leflunomide that may be useful in cancer therapy [177]. Screening for anti-epilepsy medications for Drave syndrome has shown that lorcaserin reduces the severity of seizures [178].

One of the first studies to successfully use a genetically encoded fluorescent sensor for drug screening in zebrafish demonstrated the ample opportunities this method provides for finding drugs for complex central nervous system disorders [179]. In the paper it was shown that with the GCaMP5G Ca^2+^ sensor it is possible to acquire a real-time whole-brain image of neuronal activity at the level of cellular resolution in live, unanesthetized zebrafish. For that purpose, a high-throughput autonomous system for orienting zebrafish and recording brain-wide neuronal activity was developed. Subsequent classification of neuronal activity provides an opportunity for drug screening based on drug induced brain patterns. This study is of particular interest in light of the development of new γ-aminobutyric acid [180], dopamine [181], and norepinephrine [182] fluorescent sensors that open up new possibilities for drug screening and research.

Tissue and cell specific distribution of sensors can provide off-target drug activity readout. For example, cardiotoxicity, an important drug safety concern, could potentially be monitored with voltage or Ca^2+^ sensors targeted to cardiomyocytes [183]. A wide color pallet of sensors can additionally provide multiparameter readout without spatial separation in one cell type or in a specific tissue. In such a way green DA1m dopamine sensor could be used in combination with red jRCaMP1a Ca^2+^ sensor for simultaneous registration of neuronal activity and dopamine release in brain circuits [181,184].

The simplicity of creating human disease models in zebrafish and the expanding array of fluorescent sensors will make a significant contribution to future chemical screens.

**Table 1:** Fluorescent sensors successfully tested and applied for basic research in zebrafish.

Sensor	Sensor Specificity	Sensor Location	Ref
GCAMP3	Ca^2+^	lateral line hair cells	[185]
GCAMP5G	Ca^2+^	retina, tectum	[186]
RCaMP	Ca^2+^	Trigeminal Neurons	[184]
GCAMP6	Ca^2+^	neurons	[92]
		Mauthner neurons	[187]
		ubiquitous	[188]
		neurons	[189]
sPAGCaMP6f	Ca^2+^	motor neuron, Müller glia, retinal ganglion cells	[190]
jRGECO1a	Ca^2+^	neurons	[191]
K-GECO1	Ca^2+^	spinal sensory neurons	[192]
mNG-GECO1	Ca^2+^	neurons	[193]
FGCaMP7	Ca^2+^	neurons	[194]
CaViar	Ca^2+^ and voltage (but dim)	miocardium	[91]
CaMPARI2	activated neuronal ensembles	neurons	[195]
Sypher3s	pH	ubiquitous	[196]
pHluorin	pH	Retinal Horizontal Cell–Cone Synapse	[197]
syPhy	pH	lateral line hair cells	[198]
Bongwoorie	voltage	neurons	[199]
ASAP1	voltage	neurons	[200]
zArchon1	voltage	neurons	[201]
roGFP2-Orp1	H_2_O_2_	endothelial cells and cardiomyocytes	[202]
Hyper	H_2_O_2_	ubiquitous	[203]
HyPer3	H_2_O_2_	ubiquitous	[204]
HyperRed	H_2_O_2_	ubiquitous	[205]
Hyper7	H_2_O_2_	ubiquitous	[206]
Grx1-roGFP2	Glutathione redox state	endothelial cells and cardiomyocytes	[202]
iGluSnFR	extracellular glutamate	optic tectum	[207]
		glial cells throughout the nervous system	[208]
		hair cell ribbon	[209]
DA1m	extracellular dophamine	neurons	[181]
iGABASnFR	extracellular GABA	neurons of zebrafish cerebellum	[180]
GRABNE1m	extracellular norepinephrine	neurons	[182]
iNap1	NADPH	ubiquitous	[205]
SoNar	NADH/NAD+ ratio	ubiquitous	[210]
REX-YFP	NAD^+^/NADH ratio	lateral line hair cells	[211]
Caspase 3 FRET sensor	caspase 3 activity	ubiquitous	[212]
C3	caspase 3 activity	skin cells	[213]
Voltron	voltage	neurons	[214]

### 8.4. Perspectives on the Development of Genetically Encoded Fluorescent Biosensors Suitable for Drug HTS

Genetically encoded fluorescent sensors useful for HTS drug screenings in animal models should develop towards increasing fluorescense intensity and dynamic range to minimize data variation and background influence. Applying sensors based on the red and far-red fluorescence proteins will allow microscopy in deeper layers of the animal object with minor cell and tissue damage. Ratiometric sensors development will prevent dependency on sensors’ expression level. FLIM imaging in the drug HTS is less sensitive to the loss of donor emission intensity caused by scattering in tissues and accelerates data acquisition. Detecting lifetime donor fluorescence in the FLIM-FRET makes it possible to use FRET pairs with a poor acceptor quantum yield, which increases the variety of FP combinations for FRET. Moreover, less photostable FRET pairs could be used for FLIM-FRET sensors, since reduced excitation power and wider emission filters are needed. Background-free acquisition, high sensitivity and deep penetration into tissues characterize multiphoton excitation microscopy. This type of excitation can allow fluorescent imaging in violet and blue spectum without tissue damage. Multiphoton excitation microscopy also extends the panel of developing fluorescent sensors useful for drug HTS. Finally, the development of imaging hardware and image processing methods will also broaden the range of biological processes available to observe and target in a high-throughput mode.

## 9. Conclusions

The genetically encoded fluorescent sensors are a valuable instrument for biochemical, cytological, and physiological research. They provide a specific readout, a high spatiotemporal resolution, and a possibility to observe and distinguish fine processes in live objects in a real-time mode. Moreover, they can be used in a wide range of model systems, from cellular organoids to intact organisms. Altogether, it makes this approach very promising for drug development, especially for the HTS stage, because it eliminates drugs of low specificity and inappropriate metabolism more effectively than the assays performed on fixed objects or in vitro. To date, the genetically encoded sensors have been used in search of cell signaling, metabolism, and ion transport modulators, toxicants, stress and cell death inducers. What is more, they have been used in testing drug delivery in model organisms and drug resistance in individual patients.

The use of genetically encoded fluorescent sensors seems to have a bright future because there are many promising directions in enhancing the sensors’ robustness and sensitivity, refining the imaging techniques, and developing new model systems for HTS of drugs.

## Abbreviations

AD	Alzheimer’s disease
AKAR	A-kinase activity reporter
AP	action potential
BRET	bioluminescence resonance energy transfer
CRISPR	clustered regularly interspaced short palindromic repeats
CML	chronic myelogenous leukemia
CRM	caloric restriction mimetics
CMs	cardiomyocytes
cpYFP	circularly permuted yellow fluorescent protein
DAG	diacylglycerol
EAD	early afterdepolarization
ERKs	extracellular signal-regulated kinases
ECFP	enhanced cyan fluorescent protein
EGFR	epidermal growth factor receptor
FPs	fluorescent proteins
FLIM	fluorescence lifetime imaging
FLT	fluorescence lifetime
FRET	Förster resonance energy transfer
GPCR	G protein-coupled receptor
GEVI	genetically encoded voltage indicators
GECI	genetically encoded calcium indicators
HTS	high-throughput screening
HMGB1	high-mobility group box 1 protein
iPSCs	induced pluripotent stem cells
ICD	immunogenic cell death
JNKs	c-Jun N-terminal kinases
LDG	lactate dehydrogenase
MAPK	mitogen-activated protein kinase
NLS	nuclear localization signal
OXPHOS	oxidative phosphorylation
PIP2	phosphatidylinositol 4,5-bisphosphate
PLC	phospholipase C
PLB	phospholamban
PS1	presenilin 1
PTU	1-phenyl 2-thiourea
ROS	reactive oxygen species
RTKs	receptors of growth factors, cytokines, and hormones
SERCA2a	sarco/endoplasmic reticulum Ca2^+^ ATPase
SFCAI	switch-on fluorescence-based caspase-3-like protease activity indicator
SPB	streptavidin binding protein
ssODN	single-stranded DNA oligonucleotide
TMRM	tetramethylrhodamine, methyl ester
UGI	uracil DNA glycosylase inhibitor
VEGFR	vascular endothelial growth factor
VGCs	voltage-gated ion channels
